# Refinement and Truncation of DNA Aptamers Based on Molecular Dynamics Simulations: Computational Protocol and Experimental Validation

**DOI:** 10.1021/acs.jcim.5c00243

**Published:** 2025-04-14

**Authors:** Ana Díaz-Fernández, Carmen S. Ciudad, Natalia Díaz, Dimas Suárez, Noemí de-los-Santos-Álvarez, M. Jesús Lobo-Castañón

**Affiliations:** † Departamento de Química Física y Analítica, 16763 Universidad de Oviedo, C/Julián Clavería, 8, 33006 Oviedo, Asturias, Spain; ‡ Instituto de Investigación Sanitaria del Principado de Asturias, Av. de Roma, 33011 Oviedo, Asturias, Spain

## Abstract

Aptamers have proven useful for a wide variety of applications, such as drug delivery systems and analytical reagents for diagnosis or food safety control. Conventional aptamer selection methods typically produce sequences longer than necessary, which are optimized through a postselection trial and error process to obtain the shortest-length sequence that preserves binding affinity. Herein, we describe a general strategy to obtain the tridimensional structure of DNA aptamers using a semiautomated molecular dynamics protocol, which serves as a guide to rationally improve experimentally selected candidates. Based on this approach, we designed truncated aptamers from previously described ligands recognizing different peptides and proteins, which are 20–35% shorter than the original candidates and present similar or even improved binding affinities. Moreover, we also discriminate between energetically similar secondary structures in terms of the energetic scoring of the molecular dynamics trajectories and rationally explain the role of poly thymine spacers in the (de)­stabilization of the structure. This work demonstrates how a protocol for generating the aptamers tridimensional structure can accelerate their optimization for obtaining better analytical reagents and therapeutic agents.

## Graphical abstract

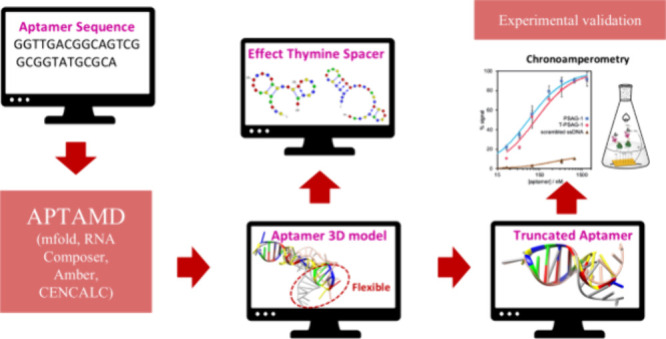

Nucleic acid aptamers are single-stranded (ss) oligonucleotides selected to bind with high affinity and selectivity a wide range of targets (e.g., metal ions, small molecules, macromolecules, viruses, and cells).^1^ They have a low molecular weight that reduces their immunogenicity, greater stability compared to traditional recognition probes such as antibodies, and a relatively simple large-scale chemical synthesis that allows rapid and reliable production and easy modification.^2^ These favorable properties explain their emerging potential as novel and flexible molecular recognition systems with applications in diagnostics, therapeutics, and biosensing.^3^

Aptamers are selected in vitro by screening large and random libraries of oligonucleotides using a procedure called SELEX (systematic evolution of ligands by exponential enrichment).^4,5^ The selected sequences combine a central randomized region of 30–80 nucleotides (nt) with fixed primer regions of ∼20-nt that are needed for amplification steps. Small random regions of 30-nt or less are more susceptible to unwanted influence from primers, while large random sequences generally require additional optimization steps to enhance their performance and applicability.^6,7^

The truncation of aptamers is a post-SELEX strategy that can improve their applicability, not only by reducing synthesis costs, but also by avoiding unspecific interactions and/or unwanted hybridization between base pairs involving the unnecessary sequences.^8,9^ Truncation has been also used to introduce the structure-switching functionality required for sensing applications,^10^ and to get useful information on primary sequences relevant for molecular recognition and target binding.^11^ In general, successful truncation proceeds by removing nonessential nucleotides from the original sequence to get a shorter molecule with the same or better binding affinity and target specificity than the original one. Thus, primer regions are normally considered as nonessential sequences in consonance with former bioinformatic analysis showing that they do not, in general, contribute to the secondary structure of aptamers.^12^ However, there are known exceptions to this general trend, where residues in the primer regions deliberately contribute to the structure and stability of the aptamer.^13^

Beyond the removal of primers, rational truncation strategies might also include additional nucleotides from the random sequence. One common approach is based on the analysis of repeated motifs in the primary sequence and/or secondary structure (2D) predictions provided by online servers.^14,15^ Assuming that conserved primary and secondary elements would be likely involved in the interaction with the target,^8^ unstructured single-stranded regions are selected for removal. This strategy has been often successful for aptamers with abundant intramolecular contacts but may fail for aptamers with weaker structures.^16^

Clearly, the availability of tridimensional (3D) models of aptamer molecules may result in more efficient truncation strategies. For example, some authors have proposed to use molecular docking tools to build approximated 3D models of an aptamer-target complex and, subsequently, remove those residues that are irrelevant in the observed binding determinants.^17^ However, it must be noted that this or similar methods require highly reliable 3D models of the aptamer and its target to identify the nonessential nucleotides for target binding. Moreover, it is equally important to preserve all residues that contribute to stabilizing the functional 3D structure regardless of their location with respect to the recognition site, including the linkers/spacers usually attached to the 5′- and 3′- ends of random sequences to immobilize aptamers or to introduce probes, as these residues may potentially influence the conformation and binding affinity of aptamers.^18,19^

Several bioinformatics tools are available for the structural prediction of single stranded nucleic acids to obtain initial 3D models of aptamers that are then typically sampled using molecular dynamics (MD) simulations.^20–24^ Based on the methodological proposals by Jeddi and Saiz,^20^ herein we present our computational protocol that relies on an extensive sampling of the conformational space of aptamers in explicit solvent using both enhanced and conventional MD simulations on the μs time scale.^25^ The protocol named APTAMD, which integrates multiple programs to generate and analyze a large variety of molecular data, is applied to predict and sample the 3D structure of DNA aptamers selected against three different targets: the 33-mer immunodominant peptide of gluten in celiac disease,^26,27^ the prostate specific antigen (PSA) glycoprotein used as serum marker in prostate cancer,^25,28,29^ and a collagen XI peptide fragment from the nonhelical C-telopeptide.^30,31^ We show first how and why to use the APTAMD protocol to better discriminate between alternative structures generated from structurally different but energetically close 2D models. Next, we discuss how to identify fragments for removal by means of clustering analysis that characterize both the well-structured regions that are most likely essential and the highly flexible segments that may be truncated to get a shorter but still functional aptamer. In addition, we study the introduction of thymine spacers in some of the selected random sequences, showing how to determine and evaluate their structural role. Importantly, all the computational predictions for the examined ssDNA sequences are experimentally validated by measuring relevant binding affinities, suggesting thus that the APTAMD protocol can be a useful tool to truncate and refine aptamers for a wide range of applications.

## Methods

### Computational Protocol

Our computational workflow for aptamer model building is summarized in Figure 1. First, the aptamer sequence is passed to the mfold program that predicts one or several secondary structures for a given temperature and ionic strength.^14^ The selected 2D models are transferred to the RNA Composer Web server^33^ to generate their initial 3D structures. We employ then our APTAMD collection of scripts to automate the application of advanced molecular modeling techniques as implemented in the AMBER suite of programs.^32^ Thus, each initial 3D model is processed by the do_aptamer_edition script to prepare the system for MD simulations as described in the Supporting Information. The do_runmd script is then used to perform a Gaussian accelerated MD (GaMD) simulation that provides an enhanced sampling of the conformational space of the aptamer,^34^ which can be particularly relevant to assess the stability of the intramolecular contacts provided by the initial 2D model and to explore different conformations for the less structured parts of the aptamer sequence. Other software tools automatize the free energy reweighing of the GaMD trajectory (see a detailed description in the Supporting Information) to get the most likely 3D model of the aptamer. The final step of the APTAMD protocol explores the equilibrium conformational space of the 3D model using conventional MD (cMD) simulations on the μs time scale. The cMD trajectories can be characterized by clustering calculations that determine the different aptamer conformations (do_cluster script) as well as by the estimation of its end-point free energy using the Molecular-Mechanics Poisson–Boltzmann Surface Area (MM-PBSA) approach (do_mmpbsa).^35^ In addition the MM-PBSA energies may be augmented with conformational entropy (*S*_conform_) calculations of the solute atoms as carried out with the CENCALC software.^36^

**Figure 1:**
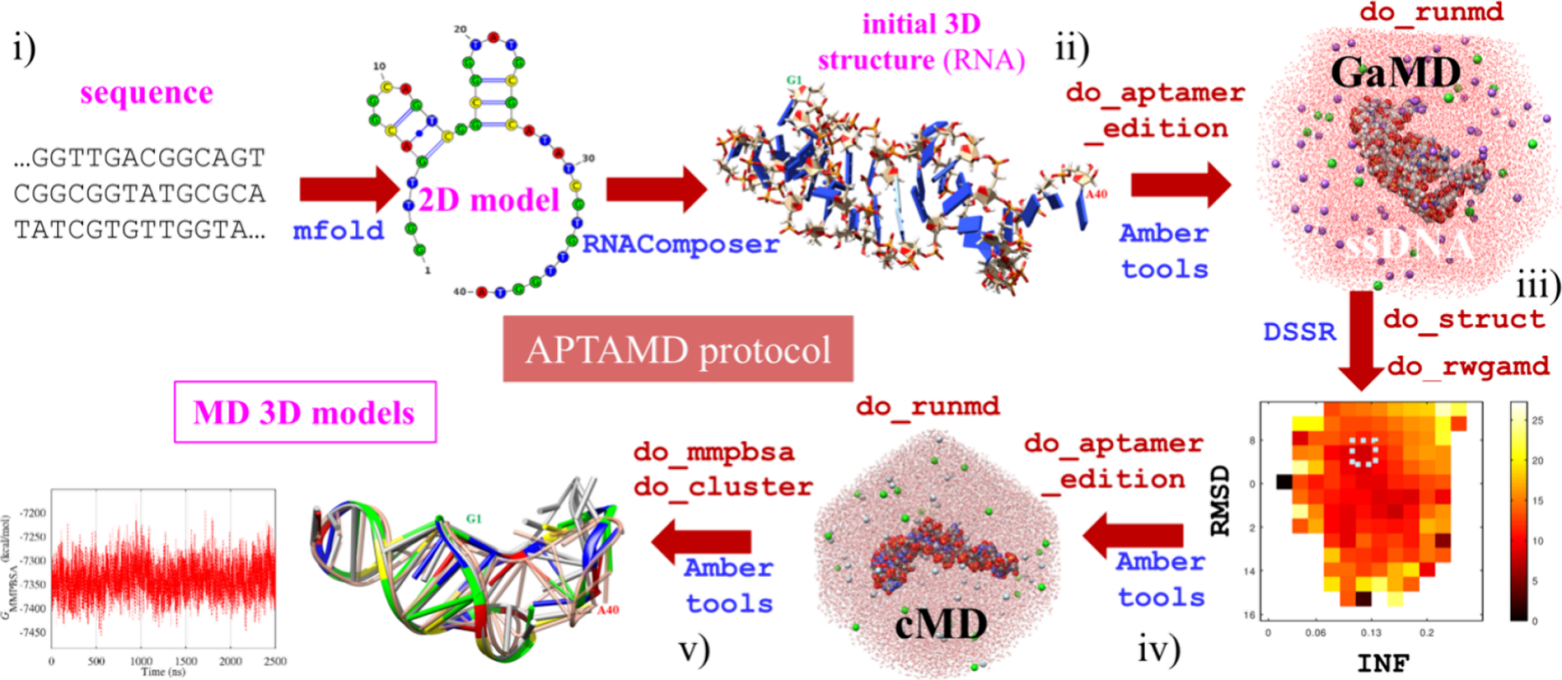
Sequential stages for building and sampling the 3D structure of a selected aptamer using the APTAMD protocol: (i) Building of initial 2D and preliminary 3D models, (ii) Gaussian-accelerated MD simulations, (iii) structural analysis and construction of free energy maps, (iv) conventional MD simulations, and (v) clustering, energy scoring and other analyses of the conventional MD trajectories.

In the Supporting Information, we briefly describe an application of APTAMD to model three DNA sequences that challenged the computational approach applied in a previous work.^20^ By comparing the APTAMD results (Figures S1–S3) with the nuclear magnetic resonance (NMR) structures deposited in the protein data bank with codes 2LO8, 1EN1, and 1NGU, we confirm that APTAMD improves former predictions by allowing ample relaxation of the systems and discriminating energetically between alternative models.

### Binding Affinity Measurements

Surface plasmon resonance (SPR) experiments and electrochemical measurements were used to characterize the binding affinities of the aptamers for their targets. Protein (PSA or streptavidin) surface modification of both Au sensor chips for SPR experiments and screen-printed Au electrodes for electrochemical measurements was performed on a mixed self-assembled monolayer (SAM) of mercaptohexanol and mercaptoundecanoic acid as previously described.^25^ For gluten aptamers assays, 50 μg·mL^–1^ biotinylated 33-mer peptide dissolved in the binding buffer (Supporting Information) was incubated for 30 min onto the streptavidin-modified chip/electrode, and then, the surface was blocked with 500 μM of biotin in the same buffer for 15 min.

Interaction with increasing concentrations of aptamers (50–5000 nM) were also performed in the respective binding buffers as indicated elsewhere.^25^ In the case of gluten aptamers assays, 0.75 μg·mL^–1^ of the conjugated streptavidin-HRP was added in the binding buffer. All the interactions were performed in an 8 μL drop at RT on the working electrode. Finally, the electrochemical measurements were carried out adding 35 μL of TMB substrate on the electrochemical cell, and after 30 s of enzymatic reaction, the reduction current of the oxidized enzymatic product was measured by chronoamperometry at −0.2 V for 1 min. The signal for each aptamer concentration corresponds to the average of the last 10 s of the measurement.

Dynabeads MyOne Streptavidin C1 were modified with biotinylated 10-mer peptide following the manufacturer instructions and the protocol previously reported.^30^ The electrochemical measurements on screen-printed carbon electrodes were carried out as described elsewhere.^31^

## Results and Discussion

### APTAMD as a Tool To Discriminate between Different Structures

Aptamer optimization mostly relies on close inspection of the secondary structure predicted by tools as mfold or NUPACK.^14,15^ These programs usually provide different 2D structures ranked by the corresponding energy scores. But when the most favorable models are energetically similar, it might be difficult to discriminate among them. This is the case discussed in the Supporting Information for the 2LO8 ssDNA considered to assess the APTAMD protocol. Another more interesting example is shown in Figure 2A for the Gli1 aptamer selected against the immunodominant peptide of gliadin.^26,27^ The best 2D mfold models present close scoring energies (−1.24 and −1.05 kcal/mol), but quite different hairpin structures. We applied our APTAMD computational protocol to both the Gli1­(mfold1) and Gli1­(mfold2) models to explore their equilibrium conformational space. The time evolution of the root-mean-squared-deviation (RMSD) in Figure 2C shows that the Gli1­(mfold1) model deviates significantly from the initial structure. However, segregation of the global RMSD into the C1-A20 and A21-T40 contributions (see Figure S4) reveals that the C1-A20 segment maintains its internal conformation, whereas the A21-T40 fragment changes during the simulation. In contrast, the RMSD plots computed for Gli1­(mfold2) confirm that this model remains structurally close to the initial structure, and it is significantly more rigid than Gli1­(mfold1). Both cMD trajectories can be considered well-equilibrated after 1.0 μs.

**Figure 2:**
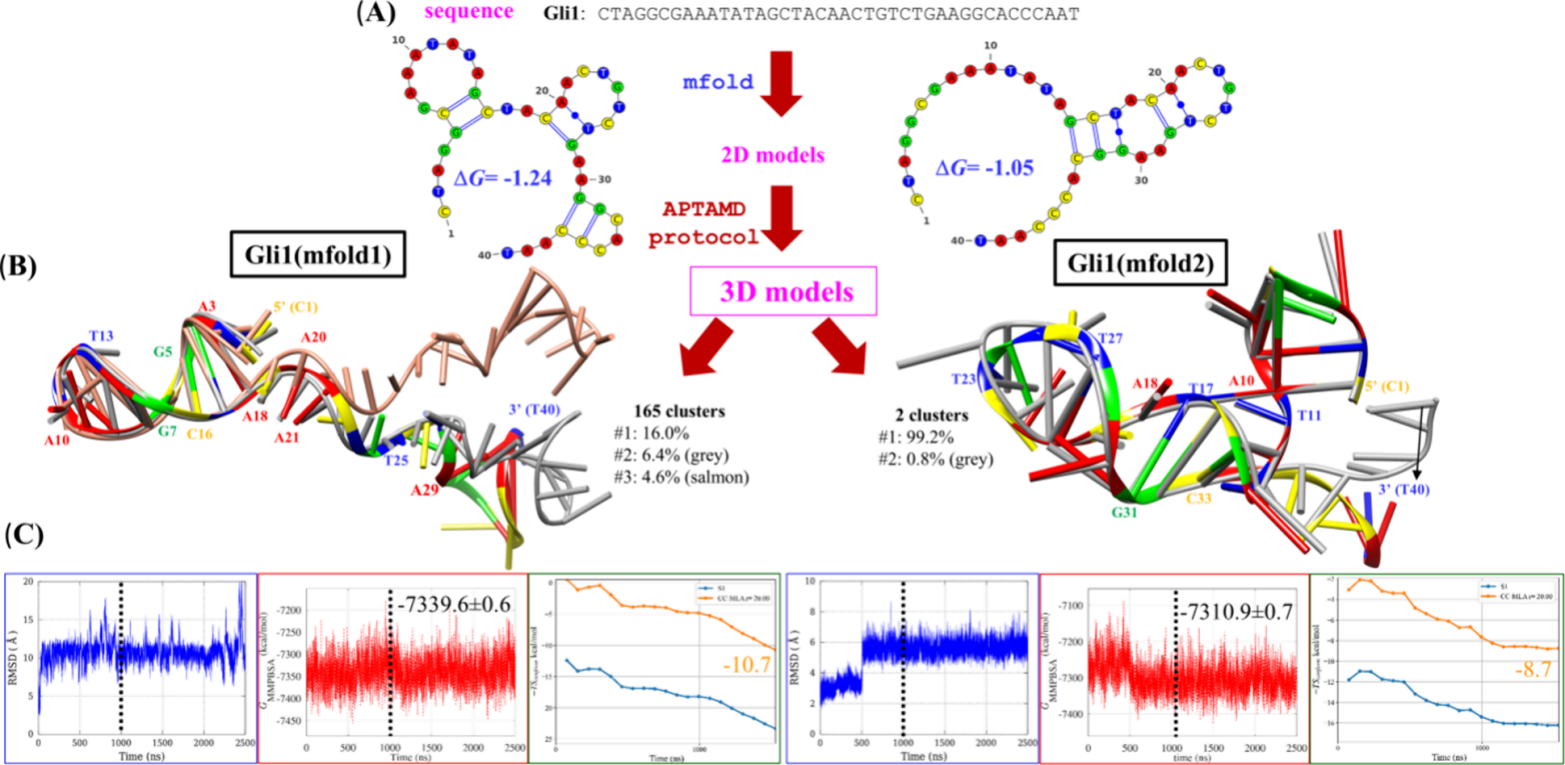
Gli1 aptamer against gluten modeled using the APTAMD protocol. (A) Sequence of Gli1 and the two most stable 2D structures predicted by mfold with scoring Δ*G* energies in kcal/mol. (B) Superposition of the three most populated cluster representatives obtained by clustering analysis performed for the last 1.5 μs of the cMD simulations considering a RMSD threshold of 5.5 Å for the backbone (i.e., sugar and phosphate) heavy atoms. (C) Time evolution of the RMSD computed for all the heavy atoms with respect to the initial structure and MM-PBSA energies along the cMD simulations. Convergence plot of conformational entropy for the last 1.5 μs.

To assess the relative stability of the Gli1­(mfold1) and Gli1­(mfold2) models, we computed their average MM-PBSA energies for the last 1.5 μs of the cMD simulations. The results point out that the Gli1­(mfold1) model would be ∼29 kcal/mol more stable than Gli1­(mfold2) (see Figure 2C). Conformational entropy calculations reinforce the preference for the Gli1­(mfold1) model by at least ∼2 kcal/mol. Thus, the minimal energy difference between the 2D models becomes more pronounced when comparing the stability of the cMD trajectories, showing that Gli1­(mfold1) is the most likely 3D model of the Gli1 aptamer. On the other hand, clustering calculations yielded 130 representatives for Gli1­(mfold1), confirming its larger flexibility compared to the Gli1­(mfold2) model (see Figure 2B). The Gli1­(mfold1) simulation present short (2.8–3.0 Å) and abundant (90–100%) base-pairing H-bond contacts that stabilize an enlarged stem in the first hairpin structure predicted by mfold (see G4···T17 and G7···A14 additional contacts in Table S3). The rest of the molecule adopts a more extended helical conformation with stacking interactions between consecutive bases from C19 to C26 (see Table S4) that is highly mobile. This difference in the structure and dynamics of the two parts of the Gli1 molecule in the most favored tridimensional model would be relevant for truncating purposes.

To further explore the applicability of the APTAMD protocol to discriminate between energetically close secondary structures, we considered the D1 aptamer selected for binding a collagen XI peptide fragment.^30^ For this sequence, mfold predicts two structurally similar 2D models with scoring energies of −5.44 and −4.61 kcal/mol that present a large and unstructured region from A27 to A40. According to the APTAMD results in Figure S5 and Table S5, the presence of abundant polar interactions all along the D1 sequence stabilizes compact and rigid 3D structures for both mfold models. But according to the average MM-PBSA energies and the limiting conformational entropy values, D1­(mfold1) is clearly the most favored model to be used in further analysis.

As a final example, we briefly retrieve the PSAG-1 aptamer selected to achieve a challenging binary recognition at the protein and glycan moieties of the PSA target.^25^ For this sequence, mfold proposes three different 2D models with scoring energies within 0.5 kcal/mol. Using a computational approach^25^ very similar to that implemented in APTAMD, we built, sampled and ranked the corresponding 3D models to unequivocally confirm PSAG-1­(mfold1) as the most favored structure (see Figure S6).

### Aptamer Truncation Based on the APTAMD Protocol

#### Two DNA Aptamers Targeting Gluten

Truncation plays a central role in the optimization of aptamers obtained by SELEX. Based on the analysis of the most repeated motifs in the SELEX sequences and the predicted secondary structures, we selected and assayed a truncation for the Gli1 and Gli4 aptamers targeting gliadin.^16^ Gli4 was truncated at the 5′ end to obtain a 19-nt sequence with residues C22–C40 that include two conserved motifs and one hairpin structure (Figure 3A). The dissociation constants obtained for the full-length and the truncated Gli4T_(22–40)_ aptamers from the corresponding binding curves using PWG (Prolamin Working Group standard) gliadin covered magnetic beads are identical within the experimental error, confirming the success of the proposed truncation. For Gli1, fragments C1-T13 and G31-T40 were removed to obtain a 17-nt truncated sequence Gli1T_(14–30)_ that conserves the first and the second motifs identified in the sequences and the corresponding hairpin structure (Figure 3B). But, in this case, the corresponding titration experiment resulted in a strongly decreased affinity toward this truncated version of Gli1.^16^ Thus, this truncation strategy based on primary and secondary structures provided highly homologue truncated aptamers (in sequence and in structure) with opposite results in terms of the affinity against gliadin.

**Figure 3:**
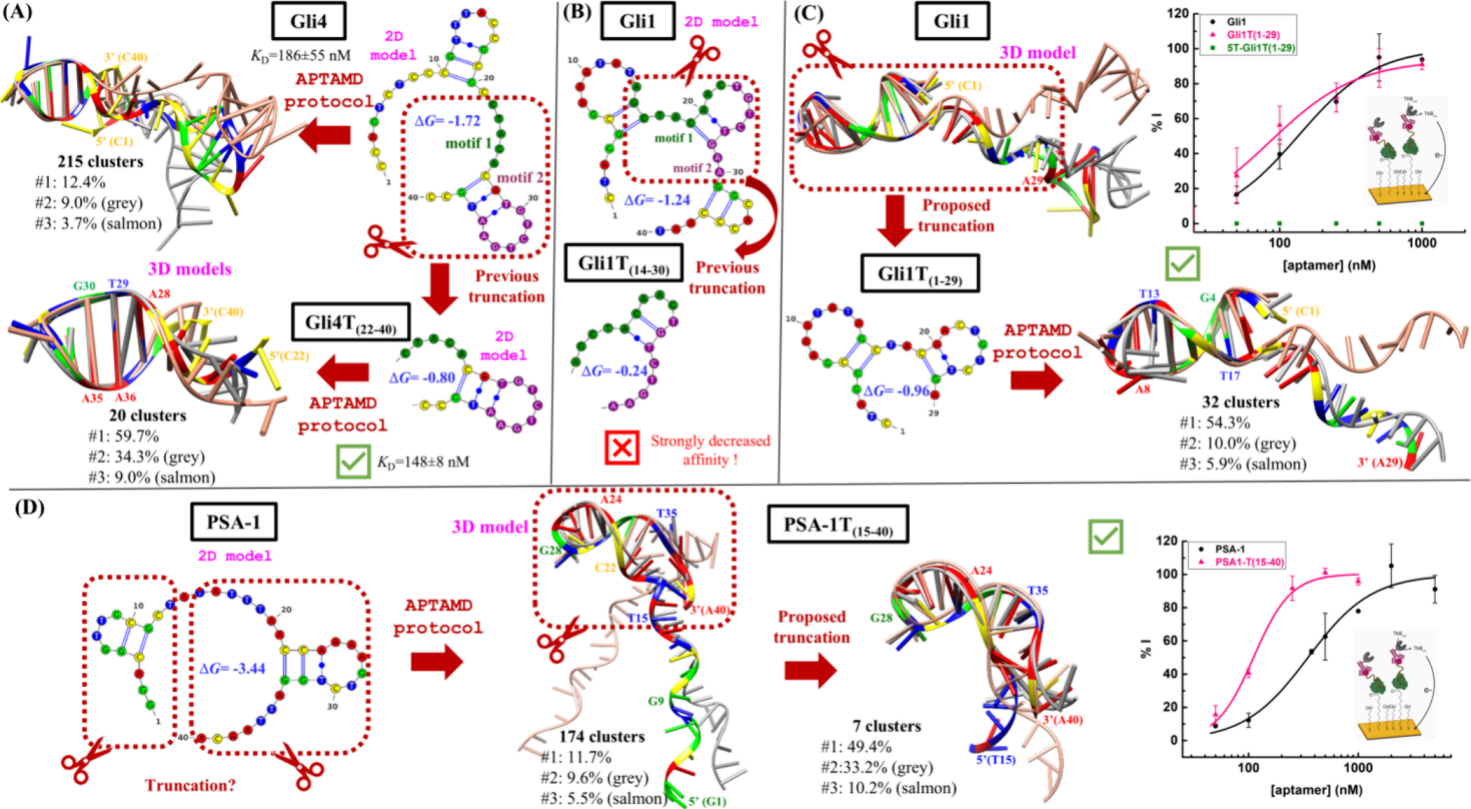
Truncation of gluten and PSA aptamers based on the APTAMD protocol. (A) Truncation of the Gli4 aptamer based on conserved motifs (dark green and dark magenta) and 2D structure predicted with the mfold Web server. The activity of Gli4T_(22–40)_ is compatible with the structure of the three most populated clusters represented as ribbons models with bases as rods and colored according to their abundance. (B) Truncation of the Gli1 aptamer based on conserved motifs (dark green and dark magenta) and 2D structure predicted with the mfold Web server. 2D model of the nonactive Gli1T_(14–30)_ truncated form. (C) Truncation of the Gli1 aptamer based on a 3D model obtained with the APTAMD protocol and represented by the three most populated clusters (ribbons models with bases as rods and colored according to their abundance). 2D and 3D (superposition of cluster representatives) models obtained for Gli1T_(1–29)_. Electrochemical binding curves obtained for Gli1 and Gli1T_(1–29)_ (square green dots correspond to the 5Ts-Gli1T_(1–29)_ aptamer discussed below). (D) Alternative truncations of PSA-1 based on the 2D model predicted with the mfold Web server. 3D model resulting from the APTAMD protocol corresponding to the superposition of the three most populated cluster representatives. Proposed truncation based on the 3D model and structure of the resulting PSA-1T_(15–40)_ truncated aptamer. Electrochemical binding curves obtained for PSA-1 and PSA-1T_(15–40)_.

To evaluate the applicability of APTAMD in the rational truncation of aptamers, we first modeled Gli4 and Gli1 starting from the most favorable mfold 2D structure (Figures S7 and S8). For Gli4, the two hairpin structures predicted by mfold in C10-G20 and C27-G38 are well preserved during the cMD simulation thanks to highly abundant base-pairing H-bond contacts at the corresponding stem regions (see Table S6). But the lack of stable interactions between residues in the first and the second half of the molecule results in wide fluctuations in their relative position as shown by the clustering results in Figure 3A. This quasi-independent dynamical evolution of the C1–C21 and C22–C40 moieties of Gli4 is in good agreement with the activity observed for the Gli4T_(22–40)_ truncated variant. In addition, mfold predicts that the truncated Gli4T_(22–40)_ sequence conserves exactly the same hairpin characterized in that region for the full-length sequence, and the cMD simulation of Gli4T_(22–40)_ confirms the enhanced structural stability of the truncated aptamer (Figure 3A).

The simulation of the most favored Gli1­(mfold1) model only preserves the first hairpin comprising residues G4-T17. The Gli1T_(14–30)_ truncation assayed in our previous work removes most of the residues in this preserved hairpin, thus explaining the loss of activity in the binding experiments. Based on the sampled Gli1­(mfold1) model and assuming that the binding site is probably located in the structured region of the aptamer, functional truncations of Gli1 should only remove residues located at the highly mobile 3′-end. In this work, we explore the effect of removing the last A30-T40 residues to give the Gli1T_(1–29)_ truncated version. According to mfold results, Gli1T_(1–29)_ presents secondary structures that conserve the stable hairpin in the full-length Gli1 aptamer (Figure S7). Starting from a representative structure from the most populated cluster obtained for the Gli1­(mfold1) simulation, we built the new truncated version of Gli1 to run the corresponding cMD simulation. The analysis of the Gli1T_(1–29)_ trajectory confirms that the main structural features of the Gli1­(mfold1) model are not perturbed by the selected truncation (i.e., all the intramolecular contacts in Tables S3 and S4 are equally abundant, resulting in a relatively rigid molecule according to the cluster results in Figure 3), so it is expected that the Gli1T_(1–29)_ aptamer remains active against gliadin.

We challenged our 3D models by performing new binding experiments with Gli1 and the truncated Gli1T_(1–29)_ aptamers. According to the electrochemical binding curve performed on 33-mer peptide-modified screen-printed Au electrodes (SPAuE), the truncated aptamer binds to the target with a similar affinity to the nontruncated one (Figure 3C and Table 1). SPR experiments, carried out in a configuration identical to the electrochemical one, also confirm the binding capability of Gli1T_(1–29)_ (Table 1). Thus, the Gli1­(mfold1) simulation provides reliable information on the 3D structure and dynamics of the Gli1 aptamer. It also emerges that truncation based on the extensive sampling of 3D structures would provide a meaningful strategy to get shorter and functional aptamers.

**Table 1:** *K*_D_ Values (nM) Obtained from Electrochemical and SPR Measurements

aptamer	chronoamperometry	SPR
PSA-1	348 ± 58	125 ± 21
PSA-1T_(15–40)_	111 ± 9	130 ± 25
PSAG-1	72 ± 8^a^	1.9 ± 0.2^a^
PSAG-1T_(1–32)_	121 ± 25^a^	not available
5Ts-24nt-3Ts	152 ± 9	232 ± 4
5Ts-24nt	460 ± 64	545 ± 24
Gli1	136 ± 19	155 ± 11
5Ts-Gli1T_(1–29)_	no binding	no binding
Gli1T_(1–29)_	86 ± 12	71 ± 14
B4	147 ± 43	not available
B4T_(11–40)_	44 ± 10	not available

a From ref 25.

#### DNA Aptamers Targeting Prostate-Specific Antigen

Truncation of the PSA-1 aptamer developed to detect aberrant glycosylation patterns for improved prostate cancer diagnosis was also analyzed based on the models provided by the APTAMD protocol.^29^ For this aptamer, mfold predicts a secondary structure with two small hairpin regions located in the first and in the second halves of the sequence (Figure 3D). The first hairpin is lost during the cMD simulation (see RMSDs in Figure S9 and polar contacts in Table S8), while the second hairpin is preserved thanks to the structural stability (i.e., 97–100% abundance) of the three canonical base pairs identified in the initial 2D model. Clustering calculations confirmed PSA-1 as a highly flexible molecule, with the first 15 nucleotides adopting an extended conformation that reorients with respect to the conserved hairpin (Figure 3D). This conformational freedom, resulting from the lack of major interactions between both regions (Tables S8 and S9), suggests that nucleotides at the 5′-end can be truncated without perturbing the structure and activity of the rest of the molecule.

We selected a truncated variant of PSA-1 including residues T15-A40 from the original sequence that presents the same secondary structure predicted for the corresponding fragment in the full-length sequence (Figure S10). PSA-1T_(15–40)_ was built by removing residues G1-A14 in the main cluster representative obtained for the complete aptamer and sampled by means of a new cMD simulation. The results confirm that the hairpin region in PSA-1T_(15–40)_ remains structurally stable and, therefore, the truncated form would remain active against PSA. Electrochemical measurements on PSA-immobilized SPAuE challenged to increasing aptamer concentrations provided the binding curves in Figure 3D and *K*_D_ values of 348 ± 58 nM for PSA-1 and 111 ± 9 nM for PSA-1T_(15–40)_. SPR measurements corroborate that the affinity of the truncated aptamer is at least as good as that of the full aptamer within the experimental error (see Table 1). Thus, the truncated PSA-1T_(15–40)_ aptamer remains active to bind PSA with an affinity similar to or greater than that of the full-length sequence.

PSAG-1 is another example of aptamer targeting PSA that was previously studied by us to get a functional truncated version.^25^ According to the MD-based model of PSAG-1 (Figure S11), the last 8 residues at the highly flexible 3′-end could be truncated to get PSAG-1T_(1–32)_. The simulation of PSAG-1T_(1–32)_ shows improved polar contacts in the stem region that results in a more stable hairpin. Affinity studies provided a *K*_D_ value of 121 ± 25 nM for PSAG-1T_(1–32)_, which is only 1.7 times higher than the one obtained for the full-length aptamer.^25^

#### DNA Aptamers Targeting Collagen XI Fragments

A final test for our truncation strategy considered some of the 40-nt aptamers selected against a peptide fragment from collagen XI.^30,31^ For the high affinity D1 sequence, the mfold1 compact structure in Figure S5 suggests that any truncation would have deleterious effects on its binding capabilities. For this reason, we considered the alternative B4 sequence that shares with D1 a highly prevalent conserved motif and the same affinity for the collagen XI target. For the most stable 2D model of B4 (Figure S12), the extended cMD simulation (7.5 μs) presents ample fluctuations in the RMSD values of residues G1-A20. The absence of stable contacts between residues G1-T12 and the rest of the molecule explains the large mobility exhibited by the 5′-end in cluster representatives shown in Figure S14. Thus, B4 could be truncated at the 5′-end without drastically altering the structure of the rest of the molecule.

Deletion of the first ten nucleotides in B4 does not alter the secondary structure predicted by mfold (see Figure S12). APTAMD application to the truncated B4T_(11–40)_ aptamer confirms that the overall structure is retained (Figure S14), with both hairpin motifs presenting slightly more stable contacts (Tables S5 and S10). Binding assays performed with a biotinylated 10-mer collagen peptide on magnetic beads confirmed that B4T_(11–40)_ binds with an order of magnitude better affinity than the original B4 sequence (see Table 1), being another validation of the APTAMD protocol to predict workable truncated aptamers.

### APTAMD To Analyze the Effect of Thymine Spacers

#### Savory et al. Anti-PSA Aptamer

To preserve the binding affinity of aptamers in analytical applications, separation of tags or anchoring groups from the aptamer sequence is a widespread strategy. Poly alkyl chains, triethylene glycol or PEG-based spacers have been used, but poly thymine spacers outperformed in avoiding steric hindrance and adequately exposing the aptamer.^18^ Savory et al. developed an aptamer against PSA called ΔPSap4#5 that was matured to improve its affinity using a genetic algorithm.^28^ This aptamer includes five and three thymine linkers at the 5′ and 3′ ends respectively, flanking a 24-nt random region. A single G-to-A base mutation was selected as the final generation sequence to stabilize a base pair with a thymine in the 3′ spacer. However, the role of the 5Ts spacer at the 5′-end is not apparent from the 2D structure (Figure 4A).

**Figure 4:**
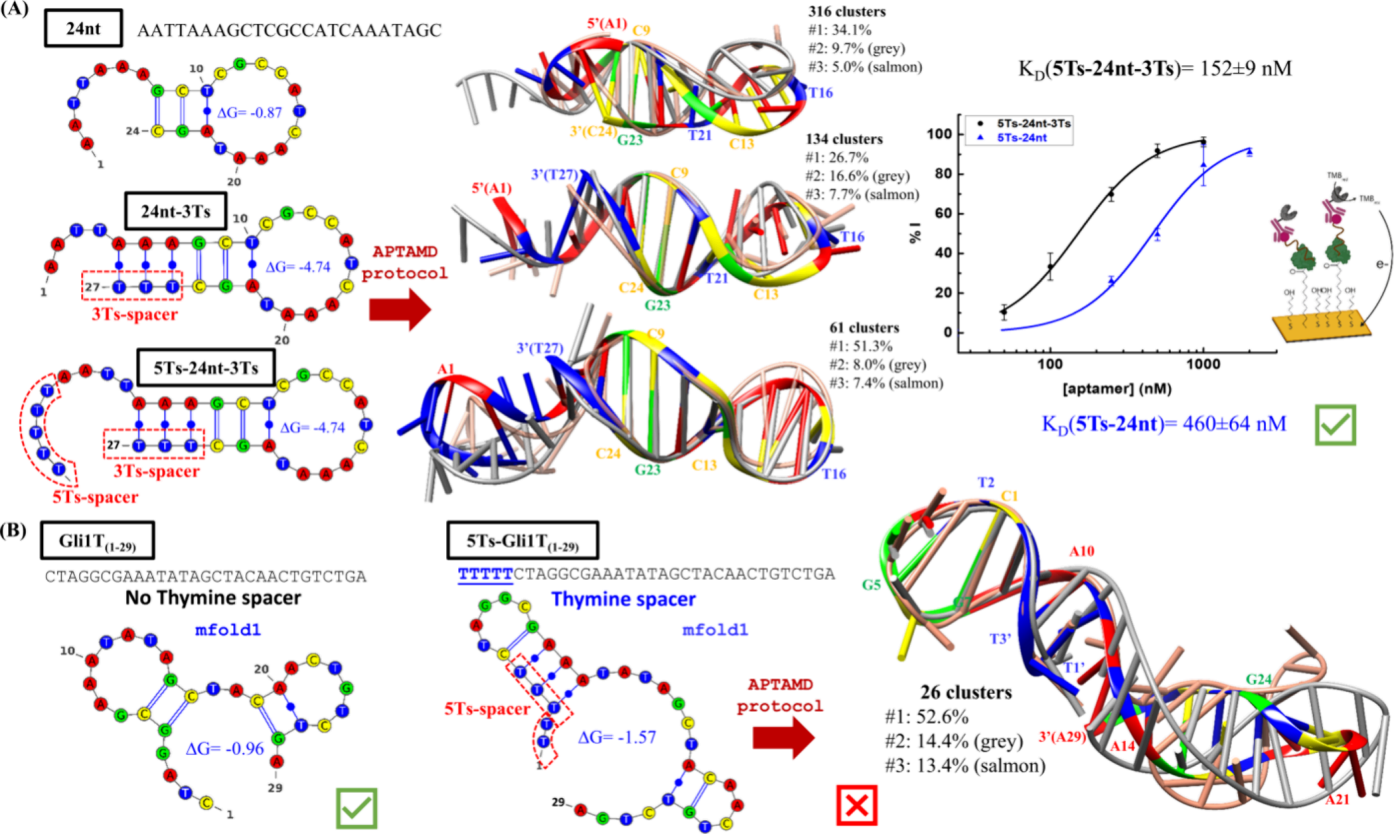
Role of polythymine spacers on aptamer structure and affinity. (A) 2D structures and scoring energies obtained for anti-PSA 24nt, 24nt-3Ts, and 5Ts-24nt-3Ts aptamers with the mfold Web server. Superposition of the three most populated cluster representatives, with percentage of abundance, performed for the last 1.5 μs of the simulations and using a RMSD threshold of 3.0 Å in the backbone heavy atoms of the common nucleotides A1-C24. Electrochemical binding curves for 5Ts-24nt and 5Ts-24nt-3Ts. (B) Most favored 2D structures obtained for Gli1T_(1–29)_ and 5Ts-Gli1T_(1–29)_ with the mfold Web server. Superposition of the three most populated cluster representatives, with percentage of abundance, performed for the last 1.5 μs of the simulations and a RMSD threshold of 5.5 Å computed for the backbone heavy atoms of the nucleotides 3–27.

To explore the relevance of the thymine spacers in the ΔPSap4#5 aptamer, we considered three different variants of its sequence: (a) the 24-nt randomized region (24nt), (b) 24nt augmented with 3Ts at the 3′-end (24nt-3Ts), and (c) 24nt with 5Ts at the 5′-end and 3Ts at the 3′-end (5Ts-24nt-3Ts). For each model, we applied the APTAMD protocol. The resulting RMSD and energy plots computed for the 24nt and 24nt-3Ts cMD trajectories (Figure S15) confirm that the 3Ts spacer stabilizes the hairpin structure due to the enlarged stem with canonical contacts (Table S11). These new interactions also reinforce canonical and noncanonical contacts at the other end of the stem (see T10···A22 and C11···T21 in Table S11). The 5 Ts at the 5′-end maintain the hairpin structure which is slightly less flexible (see 5Ts-24nt-3Ts in Figure 4A).

As a reduced hairpin is preserved during the 24nt simulation, we expected that the aptamer would remain active against PSA without the 3 Ts at the 3′-end. To test this hypothesis, we evaluated the binding ability of the 5T-24nt and 5T-24nt-3T sequences by means of electrochemical and SPR experiments. The structurally less important thymine spacer at the 5′-end was maintained for labeling purposes. *K*_D_ constants obtained from the binding curves in Figure 4A (see Table 1), confirm that removal of the 3Ts spacer at the 3′-end does not abolish the binding of the 5Ts-24nt molecule to PSA. There is a 2–4 fold reduction in its binding ability which, according to our simulations, could be related to a larger entropic penalty for complex formation in the more flexible 24nt aptamer.

#### Role of Thymine Spacers in Gluten Aptamers

The affinity of the complete Gli1 aptamer decreases when a marker molecule is incorporated at the 5′ end.^16^ For this reason, the binding ability of the new truncated Gli1T_(1–29)_ aptamer was initially evaluated including a spacer of 5 thymines at the 5′ end. However, both electrochemical and SPR experiments did not detect binding of the truncated 5Ts-Gli1T_(1–29)_ molecule to gliadin, which is in contrast with the binding capacity retained by the same truncated Gli1T_(1–29)_ sequence without the thymine spacer (see Figure 3C).

According to the APTAMD results obtained for 5Ts-Gli1T_(1–29)_, three thymines from the spacer are included in the stem of a first hairpin structure (Table S3), thus changing the structure of the aptamer (Figure 4B). Consequently, based on the 5Ts-Gli1T_(1–29)_ simulation, the lack of activity of this truncated variant of Gli1 can be ascribed to the negative structural impact of the 5 thymines at the 5′ end. In contrast, the five-thymine spacer has no effect on the structure of the Gli4T_(22–40)_ truncated aptamer according to the corresponding 2D models (Figure S8) and the activity experiments.

## Conclusions

The stability, target affinity and selectivity of aptamers, also called “chemical antibodies” due to their high targeting efficiency, depends on their structural and dynamical properties in solution. In this respect, our results highlight the necessity of improving the initial 2D and/or static 3D models of DNA aptamers to gain new and valuable understanding of their activity. To this end, we propose a particular computational protocol, APTAMD, which can be applied in a semiautomated manner to predict the structure and flexibility of an aptamer from its sequence. Based on the extensive conformational sampling in explicit solvent provided by both enhanced and conventional MD simulations on the μs time scale, APTAMD is able to (i) improve the structural predictions for challenging sequences for which NMR experimental data are available (ii) evaluate the stability of different folded states, (iii) distinguish unambiguously the structured/rigid regions from the most unstructured/flexible ones, and (iv) unveil side effects of spacers usually included for immobilization purposes or to avoid steric hindrance with attached probes. The usefulness and reliability of this protocol is supported by binding affinity measurements in different full-length and truncated aptamers. Thus, our experiments confirm that those terminal regions that are highly mobile during the simulations can be safely removed and that thymine-based spacers can influence aptamer affinity in consonance with their structural effects as revealed by the simulations. Altogether, our results support the reliability of the 3D models generated by the APTAMD protocol and their relevance for the post-SELEX optimization of aptamer sequences. Future efforts on the rational truncation of experimentally selected sequences will further assess the effectiveness of APTAMD and/or that of other approaches based on extensive MD sampling to build realistic aptamer models.

## Supplementary Material



## Data Availability

The scripts developed in this work are available on GitHub at https://github.com/dimassuarez/APTAMD. The CENCALC software for conformational entropy calculations is available at https://github.com/dimassuarez/cencalc_quicksort. The input files used/necessary for the MD simulations and restart coordinate files can be downloaded from 10.5281/zenodo.14809306.

## Notes

Detailed description of the APTAMD computational protocol and the settings selected for the simulations; test calculations for aptamers with NMR structures; reagents, equipment, and binding curve analysis; plots with the time evolution of RMSD and G_MM‑PBSA_ values, analysis of the most abundant polar and nonpolar intramolecular contacts, summary of the results obtained for aptamers D1 and PSAG-1, 2D structures predicted by mfold, and superposition of cluster representatives (PDF)

## References

[1] Cai S., Yan J., Xiong H., Liu Y., Peng D., Liu Z. (2018). Investigations
on the interface of nucleic acid aptamers and binding targets. Analyst.

[2] Wu Y., Belmonte I., Sykes K. S., Xiao Y., White R. J. (2019). Perspective
on the Future Role of Aptamers in Analytical Chemistry. Anal. Chem..

[3] Li L., Xu S., Yan H., Li X., Yazd H. S., Li X., Huang T., Cui C., Jiang J., Tan W. (2021). Nucleic Acid
Aptamers for Molecular Diagnostics and Therapeutics: Advances and
Perspectives. Angew. Chem., Int. Ed. Engl..

[4] Ellington A. D., Szostak J. W. (1990). In vitro selection of RNA molecules that bind specific
ligands. Nature.

[5] Tuerk C., Gold L. (1990). Systematic Evolution
of Ligands by Exponential Enrichment: RNA Ligands
to Bacteriophage T4 DNA Polymerase. Science.

[6] Kohlberger M., Gadermaier G. (2022). SELEX: Critical factors and optimization strategies
for successful aptamer selection. Biotechnol.
Appl. Biochem..

[7] Brown A., Brill J., Amini R., Nurmi C., Li Y. (2024). Development
of Better Aptamers: Structured Library Approaches, Selection Methods,
and Chemical Modifications. Angew. Chem., Int.
Ed..

[8] Gao S., Zheng X., Jiao B., Wang L. (2016). Post-SELEX optimization
of aptamers. Anal. Bioanal. Chem..

[9] Yu H., Zhu J., Shen G., Deng Y., Geng X., Wang L. (2023). Improving
aptamer performance: key factors and strategies. Microchim. Acta.

[10] Wolfe M., Cramer A., Webb S., Goorskey E., Chushak Y., Mirau P., Arroyo-Currás N., Chávez J. L. (2024). Rational
Approach to Optimizing Conformation-Switching Aptamers for Biosensing
Applications. ACS Sens..

[11] Yu H., Alkhamis O., Canoura J., Liu Y., Xiao Y. (2021). Advances and
Challenges in Small-Molecule DNA Aptamer Isolation, Characterization,
and Sensor Development. Angew. Chem., Int. Ed.
Engl..

[12] Cowperthwaite M. C., Ellington A. D. (2008). Bioinformatic analysis of the contribution of primer
sequences to aptamer structures. J. Mol. Evol..

[13] Yang K.-A., Chun H., Zhang Y., Pecic S., Nakatsuka N., Andrews A. M., Worgall T. S., Stojanovic M. N. (2017). High-Affinity
Nucleic-Acid-Based Receptors for Steroids. ACS
Chem. Biol..

[14] Zuker M. (2003). Mfold web
server for nucleic acid folding and hybridization prediction. Nucleic Acids Res..

[15] Zadeh J. N., Steenberg C. D., Bois J. S., Wolfe B. R., Pierce M. B., Khan A. R., Dirks R. M., Pierce N. A. (2011). NUPACK: Analysis
and design of nucleic acid systems. J. Comput.
Chem..

[16] Svigelj R., Dossi N., Pizzolato S., Toniolo R., Miranda-Castro R., de-los-Santos-Álvarez N., Lobo-Castañón M. J. (2020). Truncated
aptamers as selective receptors in a gluten sensor supporting direct
measurement in a deep eutectic solvent. Biosens.
Bioelectron..

[17] Ma P., Ye H., Guo H., Ma X., Yue L., Wang Z. (2022). Aptamer truncation
strategy assisted by molecular docking and sensitive detection of
T-2 toxin using SYBR Green I as a signal amplifier. Food Chem..

[18] Waybrant B., Pearce T. R., Kokkoli E. (2014). Effect of
Polyethylene Glycol, Alkyl,
and Oligonucleotide Spacers on the Binding, Secondary Structure, and
Self-Assembly of Fractalkine Binding FKN-S2 Aptamer-Amphiphiles. Langmuir.

[19] Amaya-González S., López-López L., Miranda-Castro R., de-los-Santos-Álvarez N., Miranda-Ordieres A. J., Lobo-Castañón M. J. (2015). Affinity
of aptamers binding 33-mer
gliadin peptide and gluten proteins: Influence of immobilization and
labeling tags. Anal. Chim. Acta.

[20] Jeddi I., Saiz L. (2017). Three-dimensional modeling
of single stranded DNA hairpins for aptamer-based
biosensors. Sci. Rep..

[21] Kilgour M., Liu T., Walker B. D., Ren P., Simine L. (2021). E2EDNA: Simulation
Protocol for DNA Aptamers with Ligands. J. Chem.
Inf. Model..

[22] Rodríguez
Serrano A. F., Hsing I. M. (2022). Prediction of Aptamer–Small-Molecule
Interactions Using Metastable States from Multiple Independent Molecular
Dynamics Simulations. J. Chem. Inf. Model..

[23] Sun X., Xie L., Qiu S., Li H., Zhou Y., Zhang H., Zhang Y., Zhang L., Xie T., Chen Y., Zhang L., Zhao Z., Peng T., Liu J., Wu W., Zhang L., Li J., Ye M., Tan W. (2022). Elucidation
of CKAP4-remodeled cell mechanics in driving metastasis of bladder
cancer through aptamer-based target discovery. Proc. Natl. Acad. Sci. U. S. A..

[24] Suzuki, Y. , Detailed Protocol for Predicting 3D Structure of DNA Aptamers and Performing In Silico Docking Calculations. In Rheumatoid Arthritis. Methods in Molecular Biology, Liu, S. , Ed. Springer US: New York, 2024; Vol. 2766, pp 139–144. 10.1007/978-1-0716-3682-4_14 38270873

[25] Díaz-Fernández A., Miranda-Castro R., Díaz N., Suárez D., de-los-Santos-Álvarez N., Lobo-Castañón M. J. (2020). Aptamers
targeting protein-specific glycosylation in tumor biomarkers: general
selection, characterization, and structural modeling. Chem. Sci..

[26] Amaya-González S., de-los-Santos-Álvarez N., Miranda-Ordieres A. J., Lobo-Castañón M. J. (2014). Aptamer
binding to celiac disease-triggering
hydrophobic proteins: a sensitive gluten detection approach. Anal. Chem..

[27] Svigelj R., Dossi N., Toniolo R., Miranda-Castro R., de-los-Santos-Álvarez N., Lobo-Castañón M. J. (2018). Selection
of Anti-gluten DNA Aptamers in a Deep Eutectic Solvent. Angew. Chem., Int. Ed. Engl..

[28] Savory N., Abe K., Sode K., Ikebukuro K. (2010). Selection of DNA aptamer against
prostate specific antigen using a genetic algorithm and application
to sensing. Biosens. Bioelectron..

[29] Díaz-Fernández A., Miranda-Castro R., de-los-Santos-Álvarez N., Rodríguez E. F., Lobo-Castañón M. J. (2019). Focusing
aptamer selection on the glycan structure of prostate-specific antigen:
Toward more specific detection of prostate cancer. Biosensors Bioelectron..

[30] Lorenzo-Gómez R., Miranda-Castro R., de-los-Toyos J. R., de-los-Santos-Álvarez N., Lobo-Castañón M. J. (2022). Aptamers targeting a tumor-associated
extracellular matrix component: The human mature collagen XIα1. Anal. Chim. Acta.

[31] Lorenzo-Gómez R., Casero-Álvarez A., Miranda-Castro R., García-Ocaña M., de-los-Toyos J. R., de-los-Santos-Alvarez N., Lobo-Castañón M. J. (2022). A competitive
assay for the detection of a 16-mer peptide from α1 chain of
human collagen XI. Talanta.

[32] Case D. A., Cheatham T. E., Darden T., Gohlke H., Luo R., Merz K. M., Onufriev A., Simmerling C., Wang B., Woods R. J. (2005). The Amber biomolecular simulation
programs. J. Comput. Chem..

[33] Popenda M., Szachniuk M., Antczak M., Purzycka K. J., Lukasiak P., Bartol N., Blazewicz J., Adamiak R. W. (2012). Automated 3D structure
composition for large RNAs. Nucleic Acids Res..

[34] Miao Y., Feher V. A., McCammon J. A. (2015). Gaussian
Accelerated Molecular Dynamics:
Unconstrained Enhanced Sampling and Free Energy Calculation. J. Chem. Theory Comput..

[35] Miller B. R., McGee T. D., Swails J. M., Homeyer N., Gohlke H., Roitberg A. E. (2012). MMPBSA.py: An Efficient
Program for End-State Free Energy Calculations. J. Chem. Theory Comput..

[36] Díaz N., Suárez D. (2022). Toward Reliable
and Insightful Entropy Calculations
on Flexible Molecules. J. Chem. Theory Comput..

