# Supplementary material for: Refinement and Truncation of DNA Aptamers Based on Molecular Dynamics Simulations: Computational Protocol and Experimental Validation
Source: J Chem Inf Model. 2025 Apr 14;65(8):4128–36. doi: 10.1021/acs.jcim.5c00243 (PMC12261273; doi:10.1021/acs.jcim.5c00243)
Supplement: Supplementary file 1 [file ci5c00243_si_001.pdf]

Refinement and Truncation of DNA Aptamers based on Molecular Dynamics Simulations: Computational Protocol and Experimental Validation

Ana Díaz-Fernández,[a,b] Carmen S. Ciudad,[a] Natalia Díaz,[*a] Dimas Suárez,[a] Noemí de-los-Santos-Álvarez,[a,b] and M. Jesús Lobo-Castañón[a,b]

[a] Departamento de Química Física y Analítica. Universidad de Oviedo, Av. Julián Clavería 8, 33006 Oviedo –Spain. E-mail: diazfnatalia@uniovi.es

[b] Instituto de Investigación Sanitaria del Principado de Asturias, Avenida de Roma, 33011 Oviedo- Spain

SUPORTING INFORMATION

Table of contents

1. APTAMD computational protocol for aptamer model building S3

1.1 Initial models: From 2D to 3D molecular edition S3

1.2 Enhanced sampling of initial 3D models: GaMD simulations S3

1.3 Selection of the most-likely GaMD conformers: Structural descriptors and free energy reweighting S4

1.4 Conventional MD: Characterizing the equilibrium properties of the aptamer models S5

1.5 Validation of the APTAMD protocol: Comparison with NMR structures S8

2. Reagents with Table S1. .... S12

3. Equipment. S13

4. Binding curves analysis with Table S2. .... S13

5. References. S14

6. Tables S3-S12 and Figures S4-S15 .... S16

1- APTAMD computational protocol for aptamer model building

The collection of scripts developed in this work to build the 3D models of aptamer molecules, which automate molecular edition, simulation and analysis tasks, is available on GitHub at https://github.com/dimassuarez/APTAMD .

1.1- Initial models: From 2D to 3D molecular edition

For a given aptamer sequence, its secondary structure is predicted using the mfold algorithm[1] that builds a series of secondary structures combining loop and stack motifs and ranks them in terms of thermodynamic data for canonical base paring including nearest neighbor effects. Common settings (T of 25 °C or 37 °C and an ionic strength of 0.150-0.250 M) are chosen for the mfold runs. From the 2D mfold structure, we generate initial 3D coordinates in PDB format using the RNA Composer webserver,[2] which decomposes the secondary structure(s) provided as input into different motifs (loop, stems, etc.), selects geometries for the various fragments from the RNA FRABASE structural database[3] allowing base replacement if necessary, employs the machine translation algorithm to generate coordinates for unmatched motifs, and builds a global 3D model by performing superimposition operations and structural relaxation.

The model(s) generated by mfold/RNA-composer are processed automatically using our driver script (`do_aptamer_edition.sh`) that executes and monitors other scripts and programs of the AMBER suite (tLEaP, cpptraj and sander).[4] The `do_aptamer_edition.sh` script transforms the atom and residue names of RNA to those of DNA, removes all 2'-hydroxyl groups on the ribose sugars, adds the missing methylene in thymines and all the H atoms, and assigns the required Molecular Mechanics (MM) parameters from the parmbsc1 force field.[5, 6] using the tLEaP program. The internal geometry of the nucleobases is relaxed by 500 steps of minimization with a distance-dependent dielectric constant and freezing the phosphate backbone atoms. Finally, the minimized structure is centered in an octahedral box of TIP3P[7] water molecules that extends at least 16 Å from the solute atoms and Na^+/Cl^- counterions are added to the solvent box in order to neutralize the negative charge of the systems and provide 0.150 M ionic strength.

1.2. Enhanced Sampling of initial 3D models: *GaMD simulations*

The conformational space of the solvated aptamer is initially explored by means of a Gaussian Accelerated Molecular Dynamics (GaMD) simulation[8] in which harmonic boost potentials are used to smooth out the potential energy surface, accelerating thus transitions between low-energy configurations. To automate this computational task, we implemented a specific driver script (`do_runmd.sh`) that

controls various preparatory stages (solvent relaxation, thermalization, pressurization and GaMD equilibration) and finally launches the production phase of the GaMD simulation. In these calculations, both the CPU and GPU accelerated version[9] of the PMEMD code are employed.

The settings of the MD calculations driven by `do_runmd.sh`, which are normally employed in many biomolecular simulations, can be briefly summarized as follows. During all the simulations, temperature (300K) is controlled by a Langevin thermostat with a collision frequency of 2 ps^{-1}, the SHAKE algorithm[10] is activated to constraint all R-H bonds, periodic boundary conditions are applied to simulate a continuous system, and a non-bonded cutoff of 9.0 Å is used in combination with the Particle-Mesh-Ewald method[11] that accounts for long-range interactions. To thermalize the systems, the solvated aptamers are first minimized and then heated gradually to 300 K by means of 60 ps of constant volume (NVT) MD with a 1 fs time step. Then the density is adjusted by running 2.0 ns of constant pressure (NPT) MD with a 2 fs time step using the Monte Carlo barostat as implemented in PMEMD.

Concerning the GaMD settings, two boost potentials, which are defined by a threshold energy E and a harmonic constant k, are applied: one to the torsion energetic term and another to the total potential energetic term including waters and counterions. The upper limits of both boost potentials are set to default values (6.0 kcal/mol) while the E and k parameters are estimated in preliminary MD and GaMD runs from the average and standard deviation of potential energies. Following the recommended prescriptions in the AMBER manual, `do_runmd.sh` assigns the values of different GaMD parameters that control the equilibration and production phases as functions of the total number of atoms. By default, the production phase of a GaMD NTP simulation is extended up to 2.5 μs. Coordinates and boost potentials are saved for analysis every 2.5 ps.

1.3- Selection of the most-likely GaMD conformers: Structural descriptors and free energy reweighting

The multiple conformations of the ssDNA molecules are characterized by two structural indexes that are largely uncorrelated to each other. On one hand, the root-mean-squared-deviation (RMSD) of the heavy atoms (P, C, N, O) with respect to the initial structure. On the other hand, the interaction network fidelity (INF) index,[12] which is built from the sets of characteristic intramolecular interactions in a reference structure (S_r) and in a given snapshot (S_m). The values of S_r and S_m, are determined by the DSSR software,[13] which identifies both base pair interactions and non-pair interactions (*i.e.*, base stacking or base contacts). A single script (`do_struc.sh`) processes all the GaMD trajectory files, evaluates these and other structural descriptors using the cpptraj[14] and DSSR programs, calculates the statistical average

and standard deviations of the RMSD/INF data and shows graphically their evolution along the GaMD trajectory.

The GaMD simulation of a given aptamer is energetically reweighed in terms of the RMSD/INF coordinates to produce a 2D free energy map of the conformational space. This task is performed using the recommended approximation to the exponential average reweighting in terms of a second-order cumulant expansion.[15] Execution of the reweighing script is controlled by another specific driver script developed by us (`do_rwgamd.sh`), which assembles all the necessary data files, selects a proper number of bins along the RMSD/INF coordinates and plots the free energy (G) and the logarithm of the unweighted population ($\log p^*$) distributions over the 2D bins. Then `do_rwgamd.sh` searches the G and $\log p^*$ maps to locate the minimum free energy basin(s) with a significant population and, subsequently, selects a set of representative structures from the GaMD trajectory files that belong to the selected basins. More particularly, the best representative structures are taken among those with the lower value of the boost potential, which would be closer to those structures that populate the equilibrium conformational ensemble.

1.4- Conventional MD: Characterizing the equilibrium properties of the aptamer models

The last phase in the MD-based construction of a 3D aptamer model consists of conventional MD simulations (cMD) that provide equilibrium conformational sampling of the aptamers, followed by structural analysis (clustering) and (if necessary) energetic rankings in solution. These computational tasks are again performed with the help of specific scripts. On one hand, the most likely conformer(s) generated by the GaMD simulation is processed by `do_aptamer_edition.sh` to place the aptamer within a new octahedral box of water and Na^+/Cl^- counterions (0.150 M ionic strength) adjusted to its overall shape. Subsequently, `do_runmd.sh` performs all the required tasks to run a cMD simulation at NPT conditions using equivalent settings to those used in the precedent GaMD simulations.

Usually, the production phase of a given cMD simulation extends up to 2.5 μs or longer to ensure that the aptamer molecule relaxes and explores its equilibrium conformations in aqueous solution. Using the `do_struct.sh` and `do_mmpbsa.sh` (see below) scripts, the time evolution of structural (e.g., RMSD) and/or energetic (e.g., MMPBSA energy) descriptors can be monitored during the cMD simulations. These and other quantities normally exhibit pronounced drifts and/or wide oscillations during the first part of the trajectory (~ 0.5-1 μs) that corresponds to the "search phase". Subsequently, the properties of the system keep fluctuating around stable mean values so that the final equilibrium properties and/or clustering analysis are better evualated using only the "fully-relaxed" part of the cMD

trajectory. In case that the "search phase" is too long, it is straightforward to resume the cMD calculations.

A critical piece of information for the rational design of truncated aptamers is obtained by carrying out a clustering analysis of the coordinates of the ssDNA models along the "fully-relaxed phase". This task, which is conveniently performed using the average-linkage clustering algorithm as implemented in the cpptraj program,[14] can be done automatically using the `do_cluster.sh` driver script that organizes all the input/output data files and assigns optimal values to the clustering options. The distance metric between MD frames is calculated via best-fit coordinate RMSD using the coordinates of the heavy atoms (C, N, O, P), selecting a maximum RMSD threshold of 5.5 Å.

To assess the importance of the various nt···nt contacts during the MD simulations, we used a software program developed locally (https://zenodo.org/records/13687928). We characterized H-bond interactions among X···Y polar groups in terms of geometrical criteria (e.g., X···Y distance< 3.5 Å and X–H···Y angle > 120º). We also scored the stacking interactions among the nucleotides in terms of an empirical dispersion potential[16] evaluated over the pairs of atoms belonging to the A/C/T/G basis regardless of their backbone connectivity (*i.e.*, we do not discriminate between stacking contacts within a stem from those involving non-pairing interactions). The criteria for assessing the occurrence of dispersion interactions were: (a) the total dispersion energy is larger than 1.0 kcal/mol in absolute value; (b) the distance between the centers of mass of the two interacting groups is below 10.0 Å.

To analyze the time evolution of the potential and solvation energy and /or assess the relative stability of different equilibrated aptamer models, our computational protocol relies on the Molecular-Mechanics Poisson-Boltzmann Surface Area (MM-PBSA) method,[17] which directly approximates the free energy of a solute molecule by combining its molecular mechanics (MM) energy with estimations of its solvation energy. Many variants of the MM-PBSA approach are routinely applied in a broad range of biomolecular modeling applications and there exist several tools[18] for streamlining this type of calculations using ensembles derived from MD simulations. In our computational protocol, we use our own driver script, `do_mmpbsa.sh`, which allows to carry out MM-PBSA like calculations with multiple options. In particular, the scoring of the aptamer models is readily available by computing the conventional MM-PBSA energy of the solute atoms as:

$$G_{MM-PBSA} = E_{MM} + \Delta G_{solv}^{PB} + \Delta G_{solv}^{non-polar} \quad (1)$$

where E_{MM} is the molecular mechanics energy including the $3RT$ contribution due to six translational and rotational degrees of freedom, ΔG_{solv}^{PB} is the electrostatic solvation energy obtained from Poisson-Boltzmann calculations,[19] and $\Delta G_{solv}^{non-polar}$ is the non-polar part of solvation energy due to cavity formation and dispersion interactions between the solute and the solvent molecules.[20]

Our driver script makes extensive use of the SANDER and PBSA programs in the AMBER package to evaluate the MM-PBSA energy terms perform. It controls the input/output of multiple data files, distributes evenly all the energy and PB calculations across the available processors, performs a statistical analysis of the results, creates different plots showing the time evolution of energy components, etc. The typical MM-PBSA settings, which are determined by the default options included in `do_mmpbsa.sh`, are as follows: The E_{MM} term is calculated with no cutoff. The ΔG_{solv}^{PB} term is obtained by solving the non-linear Poisson-Boltzmann (PB) equation on a lattice with a grid spacing of 0.33 Å and using an iterative finite-difference method. The solute is represented by the atomic charges and radii taken from the AMBER representation and treated as a low dielectric medium (ε_{in}). Several values of ε_{in} (e.g., 2, 4, 8, 10, 20) can be considered to compute the PB solvation energy and the Coulombic contributions to E_{MM}. The surrounding solvent is treated as a continuum of dielectric constant ε_{out}=80 with a 1:1 electrolyte (0.150 M) distributed according to a Boltzmann weighted average of the mean electrostatic potential. The solute-solvent dielectric boundary is the contact surface between the radii of the solute atoms and the radius (1.4 Å) of a water probe molecule.

For selected cMD simulations, conformational entropies ($S_{conform}$) were calculated using the CENCALC program[21],[22] available at https://github.com/dimassuarez/cencalc_quicksort. CENCALC transforms the time series containing the values of the rotatable dihedral angles into an array of integer numbers labelling the accessible conformational states and calculates then the unidimensional probability mass functions p_i of each discretized torsion angle. The first-order $S_{conform}$ contribution is obtained as $S_{conform} = k_B \sum_i -p_i \ln p_i$. Correlation effects were taken into account using the correlation-consistent multibody local approximation (CC-MLA).[23] Entropy bias due to finite sampling was minimized by shuffling the elements of the arrays of integer numbers labelling the conformational states prior to the entropy estimations. The CC-MLA calculations were facilitated by a cutoff-dependent formulation as well as by the classification of the discretized dihedral angles into nearly-independent, weakly- and strongly-correlated subsets, which are treated by the first-order, the second-order, and the CC-MLA techniques, respectively.[22]

1.5- Validation of the APTAMD protocol: Comparison with NMR structures

To further validate the APTAMD protocol, we modeled three ssDNA sequences with hairpin-like structures resolved by Nuclear Magnetic Resonance (NMR) measurements and deposited in the Protein Data Bank (PDB) with the codes 2LO8, 1EN1, and 1NGU. These and other ssDNAs were previously examined by Jeddi&Saiz to predict their three-dimensional structure from sequence using the mfold and the Assemble 2/UCSF Chimera programs.[24] Their predicted structures for 2LO8, 1EN1, and 1NGU exhibited the largest deviation from the corresponding first NMR model within each particular sequence length, the root mean squared deviations (RMSD) computed for the DNA backbone atoms being 5.53 Å for the shortest 2LO8 sequence (10 nucleotides), 6.89 Å for 1EN1 (18 nucleotides), and 8.59 Å for 1NGU (27 nucleotides).[24]

PDB entry 2LO8 contains 18 models structurally similar with a hairpin formed by a stem with four base-pairs and including a thymine bulge (see Figure S1). For this sequence, mfold predicts four different 2D hairpin models with only one or two base pairs in the stem that result in positive scoring values. The two most favored 2D models have similar scoring energies, but only 2LO8_mfold2 is compatible with the base pairs observed in the experimental structures. We applied the APTAMD protocol to analyze both the 2LO8_mfold1 and 2LO8_mfold2 models, and the main results are displayed in Figure S1. According to the time evolution of the MM-PBSA energies during the cMD simulations, the 2LO8_mfold2 model is the preferred one. Conformational entropy calculations do not reverse this prediction. Clustering calculations and the average RMSD values (~4.0 Å) confirm that the second mfold model is also the most structurally similar to the NMR structures. Thus, this test case exemplifies how in the absence of structural information, APTAMD can discriminate among alternative 2D and/or 3D models.

Figure S1. a) Superposition of the 18 models deposited in the PDB for the 2LO8 ssDNA with RMSD values (in Å) computed for the backbone sugar and phosphate heavy atoms with respect to the first structure. **b)** Most favored 2D models predicted by mfold for the 2LO8 ssDNA sequence with ΔG scoring energies in kcal/mol, and RMSD values computed for the RNA Composer 3D models with respect to the first NMR structure. RMSD value previously published in parentheses. **c)** Time evolution of the RMSD computed with respect to the first 2LO8 NMR model with average value and standard deviation, MM-PBSA energies with average value and statistical uncertainty (in parentheses), and S_{conform} convergence plots. Average values calculated from the dashed red line. **d)** Superposition of the most abundant cluster representatives with percentage of occurrence along the cMD simulation.

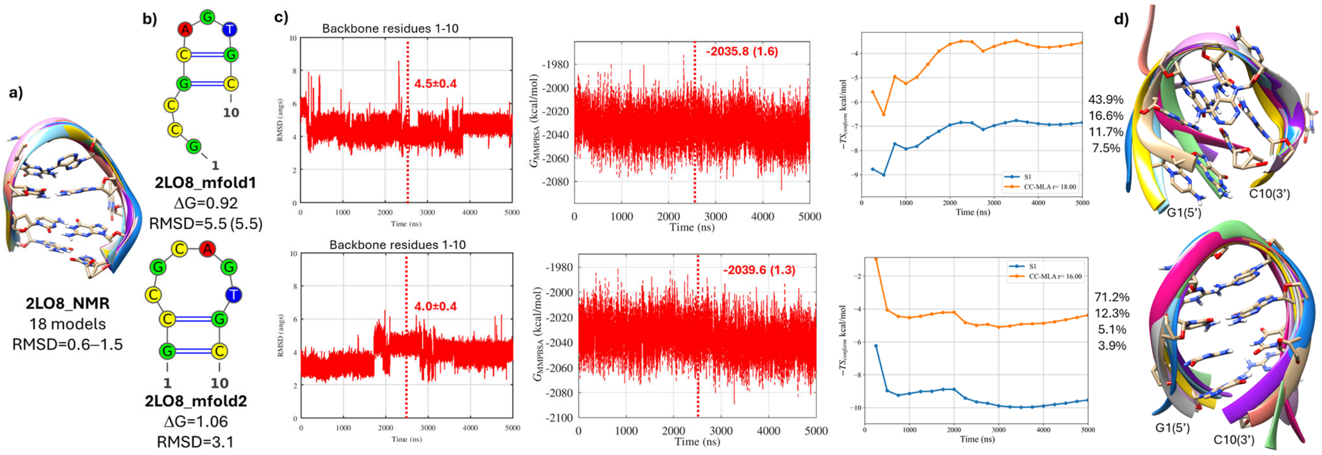

The PDB 1EN1 entry contains 20 different NMR models with backbone RMSD values in the 2.5-9.0 Å interval. This large variability results from the unstructured tail at the 3’ end that exhibits diverse orientations with respect to the more structured hairpin region. For this sequence, mfold predicts one 2D model with a stem formed by three canonical base pairs as observed in the NMR structures. The corresponding 3D model was sampled with the APTAMD protocol and the main results are shown in Figure S2. During the final cMD simulation, the hairpin structure comprising residues 1-14 is well preserved due to abundant (66-88%) hydrogen bonds in the canonical C3···G13/ C4···G12/ C5···G11 base pairs. In contrast, the last four residues at the 3’ end are highly mobile as shown by the superposition of the clustering representatives in Figure S2d. Thus, application of the APTAMD protocol helps identify and properly sample flexible regions in ssDNA sequences.

Figure S2. a) Superposition of the 20 models deposited in the PDB for the 1EN1 ssDNA with RMSD values (in Å) computed for the backbone sugar and phosphate heavy atoms with respect to the first structure. **b)** 2D model predicted by mfold for the 1EN1 sequence, Δ*G* scoring energy in kcal/mol, and RMSD value computed for the RNA Composer 3D model with respect to the first NMR structure. RMSD value previously published in parentheses. **c)** Time evolution of the RMSD computed with respect to the first 1EN1 NMR model with average value and standard deviation, MM-PBSA energies with average value and statistical uncertainty (in parentheses), and S_{conform} convergence plots. Average values calculated from the dashed red line. **d)** Superposition of the most abundant cluster representatives with percentage of occurrence along the cMD simulation.

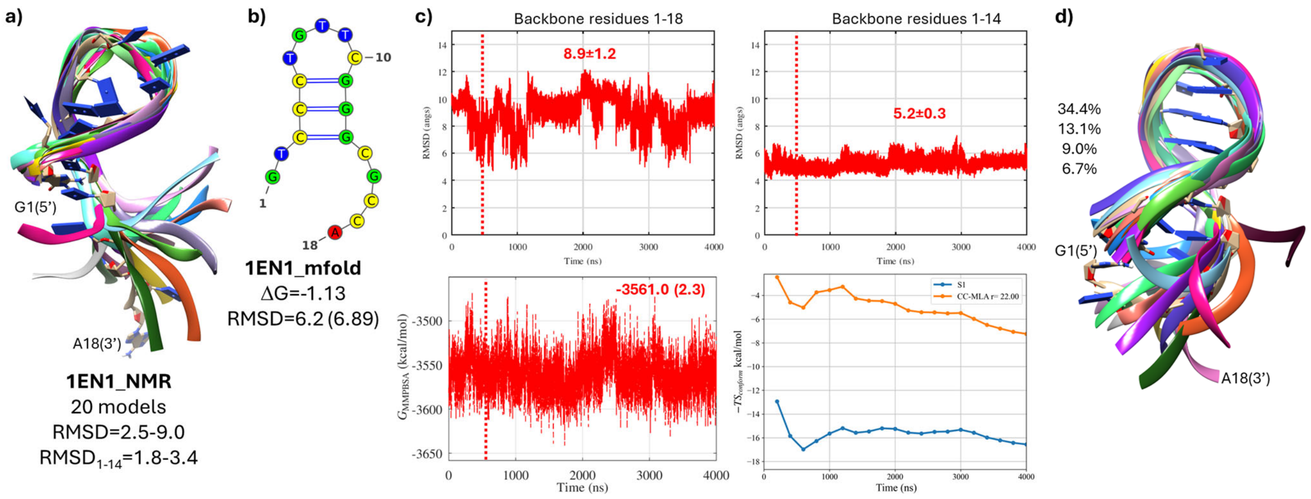

The PDB 1NGU entry contains five NMR models showing a large hairpin structure with two base mispairs at C5···A23 and C6···A22 that introduce a great deal of flexibility in and around them. For this 1NGU sequence, mfold predicts only one 2D model that is compatible with the contacts observed in the NMR structures. But the resulting 3D model presents a relatively large RMSD value (7.7 Å for our RNA Composer model and 8.6 Å for the Jeddi&Saiz model) compared to other sequences with the same length. In Figure S3, the time evolution of the RMSD and MM-PBSA energy values confirms that the final cMD simulation is well equilibrated while clustering analysis reveals that this cMD trajectory is quite close to the first NMR model. The average RMSD value computed for the last 2.0 μs of the trajectory is substantially reduced with respect to those of the initial 3D models, presenting a value (5.80±0.48Å) similar to those reported for other ssDNA sequences with 27 nucleotides studied by Jeddi&Saiz. Hence, this test case shows that extensive MD simulations can improve the agreement between experimental and computational models.

Figure S3. a) Superposition of the 5 models deposited in the PDB for the 1NGU ssDNA with RMSD values (in Å) computed for the DNA backbone atoms with respect to the first structure. **b)** 2D model predicted by mfold for the 1NGU sequence, ΔG scoring energy in kcal/mol, and RMSD value computed for the RNA Composer 3D model with respect to the first NMR structure. RMSD value previously published in parentheses. **c)** Time evolution of the RMSD computed with respect to the first 1NGU NMR model with average value and standard deviation, MM-PBSA energies with average value and statistical uncertainty (in parentheses), and S_{conform} convergence plots. Average values calculated from the dashed red line. **d)** Superposition of the most abundant cluster representatives with percentage of occurrence along the cMD simulation and superposition of the first cluster representative (red) on the first NMR model (blue) with RMSD value.

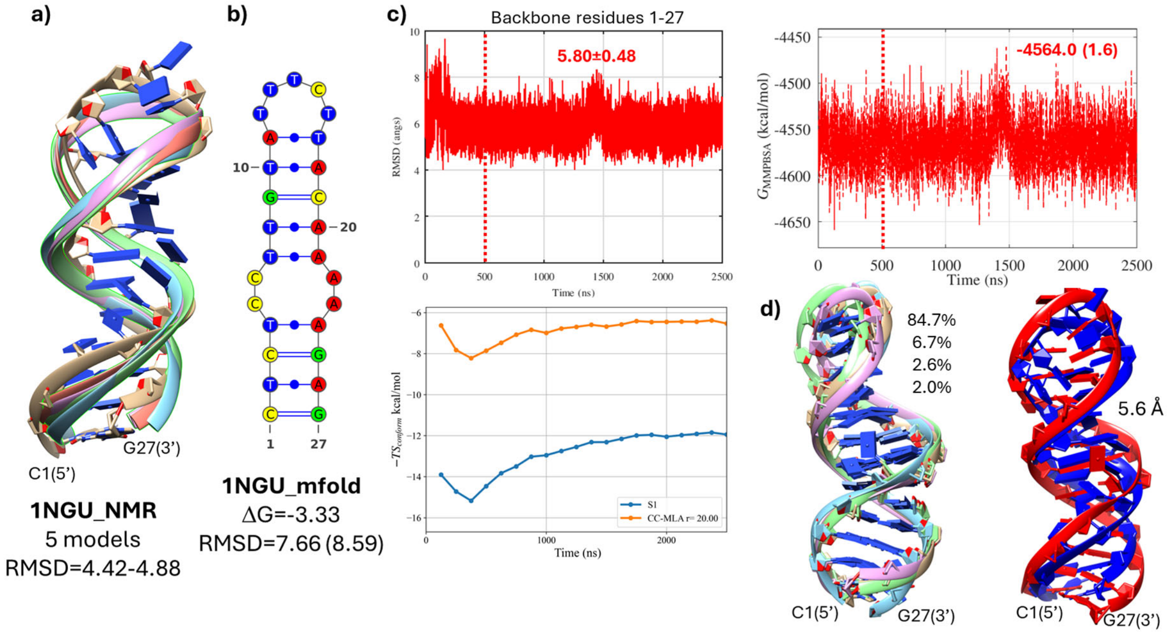

2. Reagents

Biotinylated and 6-carboxyfluorescein (6FAM) labelled DNA sequences were purchased from Metabion (Germany) and IDT (USA), respectively, as lyophilized powder with HPLC purification (Table A)

Table S1: Name and sequence of the aptamers used in the experimental part of this work.

Name	Sequence (5' → 3')
5Ts-24nt-3Ts	6FAM-TTTTT AAT TAA AGC TCG CCA TCA AAT AGC TTT
5Ts-24nt	6FAM- TTTTT AAT TAA AGC TCG CCA TCA AAT AGC
PSA-1	6FAM-TTTTT GGA CGG TTG CGC TAT ATT TAA CCA AAA GTC TGG ATT AAC A
PSA-1T$_{(15-40)}$	6FAM-TTTTT TAT TTA ACC AAA AGT CTG GAT TAA CA
Gli-1	Biotin- CTA GGC GAA ATA TAG CTA CAA CTG TCT GAA GGC ACC CAA T
5Ts-Gli1T$_{(1-29)}$	Biotin-TTTTT CTA GGC GAA ATA TAG CTA CAA CTG TCT GA
Gli1T$_{(1-29)}$	Biotin- CTA GGC GAA ATA TAG CTA CAA CTG TCT GA
B4	Biotin-TTTTT GAC GCA AGA AAT TCA GGG CAC CTG GAA CGA CGC GTC GGC T
B4T($_{11-40)}$	Biotin-TTTTT ATT CAG GGC ACC TGG, 98.3AAC GAC GCG TCG GCT

Prostate Specific Antigen (PSA) from human seminal fluid (P117-7) was purchased from BBI Solutions (UK). Biotinylated 33-mer peptide was obtained from Biomedal (Sevilla, Spain). Biotin-labelled 10-amino acid peptide from human collagen XI alpha 1 (10-mer) was supplied by Biomedal (Spain) (98.3%). Phosphate buffer 10× (PBS: 0.1 M phosphate, 1.54 M NaCl pH 7.4), 10× Tri-HCl buffer pH 7.4 and $MgCl_2$ were purchased from Merck (Spain) as molecular biology grade reagents. Sodium acetate, N-(3-dimethylaminopropyl)-N′-ethylcarbodiimide hydrochloride (EDC), N-hydroxysuccinimide (NHS), KCl, ethanolamine, 3,3´,5,5´-tetramethylbenzidine (TMB) in a format that includes H_2O_2, anti-fluorescein-peroxidase Fab fragment (Roche), streptavidin, 11-mercaptoundecanoic acid and 6-mercaptohexanol were also purchased from Merck (Spain).

Dynabeads® MyOne™ Streptavidin C1, sulfuric acid, casein 1% in PBS and TBS buffer, H_2O_2, Tween 20 (70%), and streptavidin-HRP conjugate were obtained from Thermo-Fisher Scientific (Spain). Ethanol was purchased from LabKem (Spain).

The composition of the binding buffers used for each aptamer was as follows:

- **5Ts-24nt-3Ts** and **5Ts-24nt** aptamers: 1× TBS (10 mM Tris-HCl, 150 mM NaCl, 5 mM KCl, 5 mM $MgCl_2$ pH 7.4).
- **PSA-1**, **PSA-1T$_{(15-40)}$ B4** and **B4T$_{(11-40)}$** aptamers: 1× PBS pH 7.4
- **Gli1**, **Gli1T$_{(1-29)}$**, **5Ts-Gli1T$_{(1-29)}$** aptamers: BS (50 mM Tris-HCl, 250 mM NaCl, 5 mM $MgCl_2$ pH 7.4).

Controlled temperature steps on magnetic beads (MBs) were performed in a ThermoMixer® Comfort (Eppendorf, Spain). Mixing steps at RT were carried out in a Dynabeads™ MX 12-tube Mixing Wheel (Thermo Fisher Scientific). Magnetic separation was achieved with a DynaMag™-2 magnet (Thermo Fisher Scientific).

3. Equipment

Electrochemical measurements were performed in a µAutolab II connected to a computer with NOVA 2.1 software (Metrohm, The Netherlands). Screen-printed gold electrodes (DRP-220BT) and screen-printed carbon electrodes (DRP-DSC) were obtained from Metrohm-Dropsens (Spain).

Surface plasmon resonance (SPR) measurements were carried out using an Autolab ESPRIT SPR instrument (Metrohm, The Netherlands) equipped with a two-channel cuvette and an autosampler, which is controlled by Data Acquisition Software (version 4.4). The temperature of the cell was controlled with a thermostat HaakeD1 (Germany). The SPR bare gold sensors were obtained from XanTec bioanalytics (Germany).

4. Binding curve analysis

Raw analytical data (current for electrochemical measurements and angle shift for SPR measurements) were blanked subtracted and plotted against the aptamer concentration to obtain each binding curve. All data were fitted to two models: i) the Langmuir model (eq 1) that implies a 1:1 stoichiometric interaction (single binding site), and ii) the Hill model (eq. 2), a simplification of the Adair equation that applies to molecules that exhibit interacting binding sites.

$$S_i = \frac{S_{max}[aptamer]}{K_D + [aptamer]} \qquad (2)$$

$$S_i = \frac{S_{max}^n\ [aptamer]^n}{K_D^n +\ [aptamer]^n} \qquad (3)$$

where S_i is the signal (current or angle shift for electrochemical or SPR measurements, respectively) at each aptamer concentration, S_{max} is the saturation signal, K_D is the signal at 50% of saturation and corresponds to the affinity constant, and n is the cooperativity.

The saturation signal obtained for the best fit was used to calculate the binding percentage as: $S_i / S_{max} \times 100$. The binding curves are shown in percentage for the sake of comparison. The fitting parameters of the binding curves shown in figures 2, 3 and 4 are given below.

Table S2. Fitting parameters for the electrochemical binding curves shown in the text.

Aptamer	% max	SD	K_D	SD	n	SD	r
**PSA-1**	100	7	348	58	1.5	0.3	0.9794
**PSA-1T$_{(15\text{-}40)}$**	100	4	111	9	2.6	0.4	0.9943
**5Ts-24nt-3Ts**	100	2	152	9	1.8	0.1	0.9988
**5Ts-24nt**	99	8	460	64	1.9	0.4	0.9929
**Gli1**	101	6	136	18	1.6	0.3	0.9934
**Gli1T$_{(1\text{-}29)}$**	94	5	86	12	1.4	0.3	0.9919

5. References

1. Zuker, M., Mfold web server for nucleic acid folding and hybridization prediction. *Nucleic Acids Res.* **2003,** *31* (13), 3406-15.
2. Popenda, M.; Szachniuk, M.; Antczak, M.; Purzycka, K. J.; Lukasiak, P.; Bartol, N.; Blazewicz, J.; Adamiak, R. W., Automated 3D structure composition for large RNAs. *Nucleic Acids Res.* **2012,** *40* (14), e112-e112.
3. Popenda, M.; Szachniuk, M.; Blazewicz, M.; Wasik, S.; Burke, E. K.; Blazewicz, J.; Adamiak, R. W., RNA FRABASE 2.0: an advanced web-accessible database with the capacity to search the three-dimensional fragments within RNA structures. *BMC Bioinformatics* **2010,** *11* (1), 231.
4. Case, D. A.; Cheatham, T. E., 3rd; Darden, T.; Gohlke, H.; Luo, R.; Merz, K. M., Jr.; Onufriev, A.; Simmerling, C.; Wang, B.; Woods, R. J., The Amber biomolecular simulation programs. *J. Comput. Chem.* **2005,** *26* (16), 1668-88.
5. Ivani, I.; Dans, P. D.; Noy, A.; Pérez, A.; Faustino, I.; Hospital, A.; Walther, J.; Andrio, P.; Goñi, R.; Balaceanu, A.; Portella, G.; Battistini, F.; Gelpí, J. L.; González, C.; Vendruscolo, M.; Laughton, C. A.; Harris, S. A.; Case, D. A.; Orozco, M., Parmbsc1: a refined force field for DNA simulations. *Nat. Methods* **2016,** *13* (1), 55-58.
6. Galindo-Murillo, R.; Robertson, J. C.; Zgarbová, M.; Šponer, J.; Otyepka, M.; Jurečka, P.; Cheatham, T. E., III, Assessing the Current State of Amber Force Field Modifications for DNA. *J. Chem. Theory Comput.* **2016,** *12* (8), 4114-4127.
7. Jorgensen, W. L.; Chandrasekhar, J.; Madura, J. D.; Impey, R. W.; Klein, M. L., Comparison of simple potential functions for simulating liquid water. *J. Chem. Phys.* **1983,** *79* (2), 926-935.
8. Miao, Y.; Feher, V. A.; McCammon, J. A., Gaussian Accelerated Molecular Dynamics: Unconstrained Enhanced Sampling and Free Energy Calculation. *J. Chem. Theory Comput.* **2015,** *11* (8), 3584-3595.
9. Le Grand, S.; Götz, A. W.; Walker, R. C., SPFP: Speed without compromise—A mixed precision model for GPU accelerated molecular dynamics simulations. *Comput. Phys. Commun.* **2013,** *184* (2), 374-380.
10. Ryckaert, J.-P.; Ciccotti, G.; Berendsen, H. J. C., Numerical integration of the cartesian equations of motion of a system with constraints: Molecular dynamics of n-alkanes. *J. Comput. Phys.* **1977,** *23*, 327-341.

11. Essmann, U.; Perera, L.; Berkowitz, M. L.; Darden, T.; Lee, H.; Pedersen, L. G., A smooth particle mesh Ewald method. *J. Chem. Phys.* **1995,** *103*, 8577–8593.
12. Parisien, M.; Cruz, J. A.; Westhof, E.; Major, F., New metrics for comparing and assessing discrepancies between RNA 3D structures and models. *RNA* **2009,** *15* (10), 1875-1885.
13. Lu, X. J.; Bussemaker, H. J.; Olson, W. K., DSSR: an integrated software tool for dissecting the spatial structure of RNA. *Nucleic Acids Res.* **2015,** *43* (21), e142.
14. Roe, D. R.; Cheatham, T. E., PTRAJ and CPPTRAJ: Software for Processing and Analysis of Molecular Dynamics Trajectory Data. *J. Chem. Theory Comput.* **2013,** *9* (7), 3084-3095.
15. Miao, Y.; Sinko, W.; Pierce, L.; Bucher, D.; Walker, R. C.; McCammon, J. A., Improved Reweighting of Accelerated Molecular Dynamics Simulations for Free Energy Calculation. *J. Chem. Theory Comput.* **2014,** *10* (7), 2677-2689.
16. Elstner, M.; Hobza, P.; Frauenheim, T.; Suhai, S.; Kaxiras, E., Hydrogen bonding and stacking interactions of nucleic acid base pairs: A density-functional-theory based treatment. *J. Chem. Phys.* **2001,** *114* (12), 5149-5155.
17. Wang, E.; Sun, H.; Wang, J.; Wang, Z.; Liu, H.; Zhang, J. Z. H.; Hou, T., End-Point Binding Free Energy Calculation with MM/PBSA and MM/GBSA: Strategies and Applications in Drug Design. *Chem. Rev.* **2019,** *119* (16), 9478-9508.
18. Miller, B. R., III; McGee, T. D., Jr.; Swails, J. M.; Homeyer, N.; Gohlke, H.; Roitberg, A. E., MMPBSA.py: An Efficient Program for End-State Free Energy Calculations. *J. Chem. Theory Comput.* **2012,** *8* (9), 3314-3321.
19. Sharp, K. A.; Honig, B., Calculating total electrostatic energies with the nonlinear Poisson-Boltzmann equation. *J. Phys. Chem.* **1990,** *94* (19), 7684-7692.
20. Tan, C.; Tan, Y.-H.; Luo, R., Implicit Nonpolar Solvent Models. *J. Phys. Chem. B* **2007,** *111* (42), 12263-12274.
21. Suárez, E.; Díaz, N.; Méndez, J.; Suárez, D., CENCALC: A computational tool for conformational entropy calculations from molecular simulations. *J. Comput. Chem.* **2013,** *34* (23), 2041-2054.
22. Díaz, N.; Suárez, D., Toward Reliable and Insightful Entropy Calculations on Flexible Molecules. *J. Chem. Theory Comput.* **2022,** *18* (1549-9626 (Electronic)), 7166-7178.
23. Suárez, E.; Suárez, D., Multibody Local Approximation: Application to Conformational Entropy Calculations on Biomolecules. *J. Chem. Phys.* **2012,** *137*, 084115.
24. Jeddi, I.; Saiz, L., Three-dimensional modeling of single stranded DNA hairpins for aptamer-based biosensors. *Sci. Rep.* **2017,** *7* (1), 1178.

Figure S4. Time evolution of the root mean squared deviation (RMSD in Å) computed for all the heavy atoms in selected residues with respect to the initial structure and MM-PBSA energy values (kcal/mol) along the cMD simulations run for the different Gli1 and Gli4 aptamers.

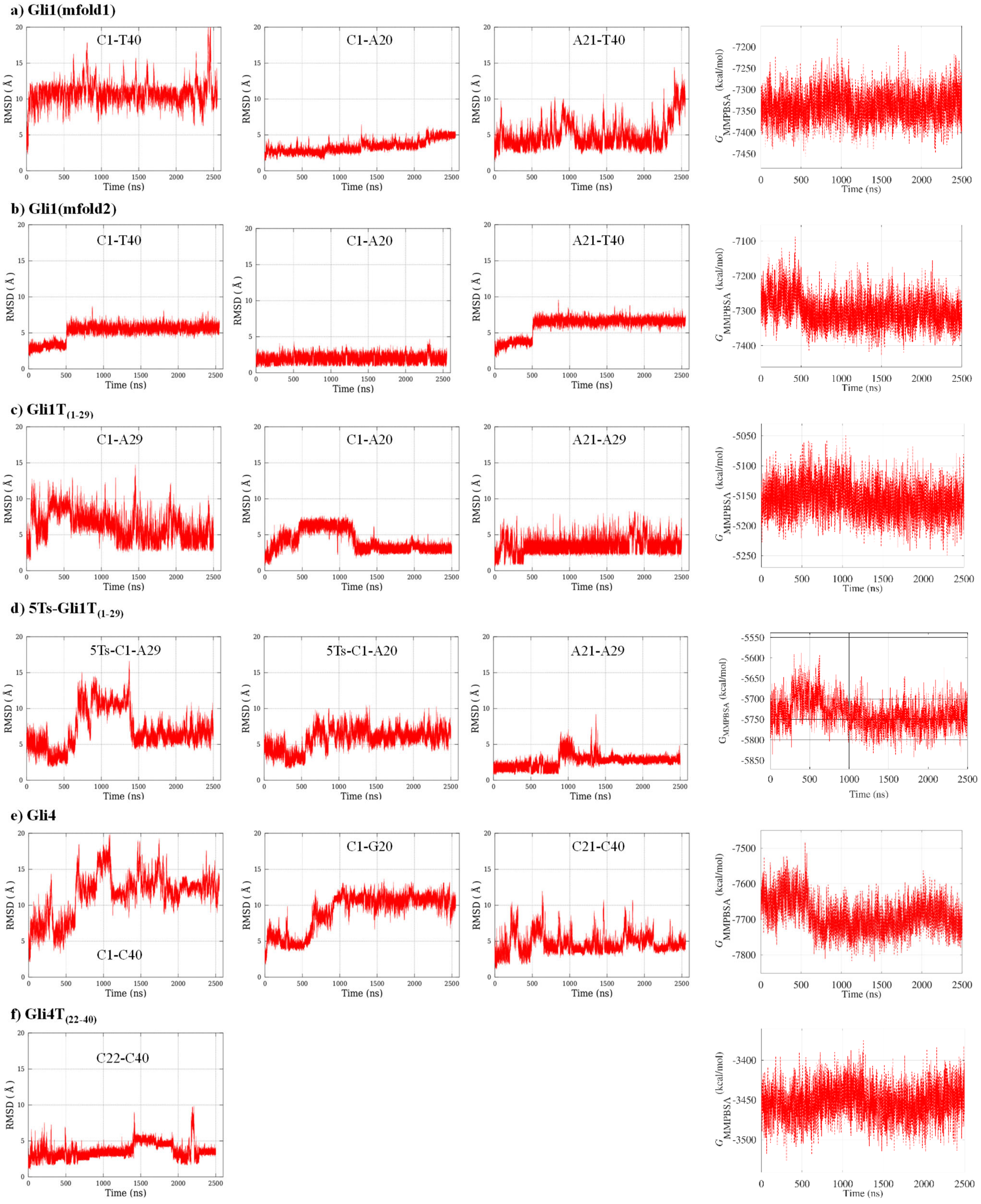

Table S3. Most abundant intramolecular H-bond contacts with percentage of occurrence (%) and average distance (d in Å) computed for the last 1.5 µs of the cMD trajectories computed for the Gli1 aptamer. The base pairing contacts in the initial 2D mfold model are in bold.

**Gli1(mfold1)**			**Gli1(mfold2)**			**Gli1T$_{(1-29)}$**			**5Ts-Gli1T$_{(1-29)}$**		
Contacts	%	d	Contacts	%	d	Contacts	%	d	Contacts	%	d
A3@N6···N3@A18	81	3.1	T2@N3···N1@A9	100	3.0	A3@N6···N3@A18	82	3.1	**T3’@N3···N1@A10**	100	3.0
G4@N1···O2@T17	100	2.8	T2@O4···N6@A9	92	3.0	G4@N1···O2@T17	100	2.8	**T3’@O4···N6@A10**	99	3.0
G4@O6···N3@T17	98	2.9	G4@OP2···N2@G7	100	2.9	G4@O6···N3@T17	98	2.9	**T4’@N3···N1@A9**	99	3.0
**G5@N1···N3@C16**	100	3.0	G4@N2···O2@C6	98	2.9	**G5@N1···N3@C16**	100	3.0	**T4’@O4···N6@A9**	98	3.0
**G5@N2···O2@C16**	100	2.9	G4@OP2···N1@G7	95	2.9	**G5@N2···O2@C16**	100	2.9	**T5’@N3···N1@A8**	100	2.9
**G5@O6···N4@C16**	99	2.9	A10@N6···OP1@T17	98	2.9	**G5@O6···N4@C16**	100	2.9	**T5’@O4···N6@A8**	98	3.0
**C6@N3···N1@G15**	100	2.9	T11@N3···O3’@C16	99	3.0	**C6@N3···N1@G15**	100	2.9	**C1@N3···N1@G7**	100	3.0
**C6@O2···N2@G15**	100	2.8	T11@N3···OP1@T17	84	3.2	**C6@O2···N2@G15**	100	2.8	**C1@O2···N2@G7**	100	2.9
**C6@N4···O6@G15**	100	2.9	A12@N3···N2@G15	99	3.0	**C6@N4···O6@G15**	100	2.9	**C1@N4···O6@G7**	99	3.0
G7@N2···N7@A14	99	3.0	A12@N6···O4’@A34	71	3.2	G7@N2···N7@A14	99	2.9	T2@O2···N1@G5	93	2.9
G7@N3···N6@A14	92	3.0	T13@OP2···N6@A34	95	2.9	G7@N3···N6@A14	94	3.0	T2@O2···N2@G5	93	3.0
G31@N3···N6@A39	81	3.0	**G15@N2···O2@C33**	100	2.9				A14@N6···O4@T27	68	2.9
G32@N1···OP2@C37	87	2.9	**G15@N1···N3@C33**	100	3.0				A14@N1···N3@T27	61	3.0
			**G15@O6···N4@C33**	100	2.9				A18@N6···N3@G24	99	3.0
			**C16@O2···N2@G32**	100	2.9				A18@N7···N2@G24	98	3.0
			**C16@N3···N1@G32**	100	3.0				A18@N6···O4’@G24	95	3.1
			**C16@N4···O6@G32**	98	3.0				A18@N6···O2@T23	92	3.0
			**T17@O2···N1@G31**	100	2.8				C19@N4···O4@T23	99	2.9
			**T17@N3···O6@G31**	93	3.0				C19@N3···N3@T23	98	3.1
			**T17@O2···N2@G31**	75	3.2				C19@O2···N4@C22	93	3.1
			A18@N3···N4@C19	95	3.1				A20@N3···N4@C22	90	3.0
			**C19@OP2···N1@G28**	97	2.8				A20@O4’···N4@C22	71	3.0
			**C19@OP2···N2@G28**	91	3.1						
			**A20@N1···N2@T27**	99	3.0						
			**A20@N6···O4@T27**	99	2.9						

Table S4. Most abundant intramolecular non-polar contacts with percentage of occurrence (%), average distance (d in Å) and interaction energy (E_{int} in kcal/mol) computed for the last 1.5 μs of the cMD trajectories computed for the Gli1 aptamer.

**Gli1(mfold1)**				**Gli1(mfold2)**				**Gli1T$_{(1-29)}$**				**5Ts-Gli1T$_{(1-29)}$**			
Contacts	%	d	E_{int}	Contacts	%	d	E_{int}	Contacts	%	d	E_{int}	Contacts	%	d	E_{int}
C1@base···base@T2	100	3.9	-3.7	C1@base···base@T2	100	4.0	-3.7	C1@base···base@T2	99	4.0	-3.6	T3'@base···base@T4'	100	4.1	-4.6
T2@base···base@A3	100	3.9	-5.6	T2@base···base@G7	100	4.0	-5.7	T2@base···base@A3	100	4.0	-5.4	T4'@base···base@T5'	100	3.9	-5.1
A3@base···base@G4	100	4.1	-5.9	A3@base···base@G4	100	3.8	-6.8	A3@base···base@G4	100	4.1	-6.0	T5'@base···base@C1	100	4.1	-4.6
G4@base···base@G5	100	4.0	-6.6	G5@base···base@A8	99	5.0	-4.0	G4@base···base@G5	100	4.0	-6.6	C1@base···base@T2	100	3.7	-5.2
G5@base···base@C6	100	3.6	-6.2	A9@base···base@A10	100	4.1	-6.0	G5@base···base@C6	100	3.6	-6.2	T2@base···base@A3	87	4.5	-4.4
C6@base···base@G7	100	3.8	-6.1	A10@base···base@T11	100	4.0	-5.5	C6@base···base@G7	100	3.7	-6.1	A3@base···base@G4	53	4.5	-5.5
G7@base···base@A8	93	6.2	-2.8	T11@base···base@A12	100	3.9	-5.7	G7@base···base@T13	85	4.3	-5.4	G5@base···base@G7	100	4.3	-5.8
G7@base···base@T13	95	6.7	-1.7	T13@base···base@G15	100	5.1	-3.6	A8@base···base@A9	70	4.3	-5.7	G7@base···base@A8	100	3.8	-6.7
A8@base···base@A14	76	4.7	-4.7	T13@base···base@C33	100	3.8	-4.3	A10@base···base@T11	98	4.4	-5.1	A8@base···base@A9	100	4.0	-6.3
A9@base···base@A10	100	5.3	-3.2	T13@base···base@A34	100	4.4	-5.2	T11@base···base@A12	100	4.2	-5.3	A9@base···base@A10	100	3.8	-6.9
A9@base···base@T11	100	5.0	-3.4	G15@base···base@C16	100	3.6	-6.4	A12@base···base@T13	100	3.6	-6.3	A10@base···base@T11	100	4.1	-5.2
A12@base···base@T13	100	3.8	-6.0	C16@base···base@T17	100	3.6	-5.2	T13@base···base@A14	82	5.6	-2.2	T11@base···base@A12	97	5.2	-3.3
T13@base···base@A14	71	7.7	-1.4	T17@base···base@A18	100	4.0	-5.2	A14@base···base@G15	100	3.9	-6.6	A14@base···base@G15	100	4.2	-5.9
A14@base···base@G15	100	3.9	-6.6	A18@base···base@G28	100	4.1	-5.9	G15@base···base@C16	100	3.8	-5.8	A14@base···base@G28	72	5.7	-3.5
G15@base···base@C16	100	3.8	-5.7	C19@base···base@G28	100	5.5	-3.2	C16@base···base@T17	100	3.7	-5.0	G15@base···base@C16	97	4.8	-4.7
C16@base···base@T17	100	3.7	-5.1	C19@base···base@G31	100	3.6	-6.2	T17@base···base@A18	100	3.7	-6.0	T17@base···base@A18	97	5.0	-3.8
T17@base···base@A18	100	3.7	-6.0	A20@base···base@A21	100	3.8	-6.7	C19@base···base@A20	100	4.0	-5.3	T17@base···base@T25	98	5.2	-3.0
C19@base···base@A20	86	4.3	-5.0	A20@base···base@G28	100	5.5	-3.6	A20@base···base@A21	100	4.0	-6.1	A18@base···base@C19	100	3.7	-5.5
A20@base···base@A21	91	4.0	-6.3	A21@base···base@G24	98	5.9	-3.2	A21@base···base@C22	99	4.2	-4.8	C19@base···base@A20	100	4.0	-5.5
A21@base···base@C22	99	4.2	-4.8	C22@base···base@T23	53	5.0	-3.2	C22@base···base@T23	98	4.1	-4.6	A20@base···base@A21	100	4.0	-6.2
C22@base···base@T23	98	4.2	-4.5	G24@base···base@T25	92	5.2	-3.8	T23@base···base@G24	99	4.1	-5.5	C22@base···base@T23	100	3.7	-5.4
T23@base···base@G24	96	4.5	-5.0	T25@base···base@C26	99	4.5	-3.7	G24@base···base@T25	100	3.7	-6.2	T23@base···base@G24	100	3.8	-5.8
G24@base···base@T25	100	3.7	-6.2	T27@base···base@G28	100	3.9	-5.4	T25@base···base@C26	95	4.0	-4.5	T25@base···base@C26	96	4.5	-3.8
T25@base···base@C26	99	4.4	-4.2	A29@base···base@A30	100	3.9	-6.4	C26@base···base@T27	99	4.0	-4.8	C26@base···base@T27	99	3.9	-4.9

Table S4. (cont.)

**Gli1(mfold1)**				**Gli1(mfold2)**				**Gli1T$_{(1-29)}$**				**5Ts-Gli1T$_{(1-29)}$**			
C26@base···base@G28	100	5.1	-3.2	G31@base···base@G32	100	3.8	-7.2	T27@base···base@G28	99	4.1	-5.4	T27@base···base@G28	100	4.0	-5.5
G28@base···base@A29	99	4.0	-6.5	G32@base···base@C33	100	3.7	-6.2								
A29@base···base@A30	100	4.0	-6.2	A34@base···base@C35	100	3.7	-6.1								
A30@base···base@G31	100	4.0	-6.2	C35@base···base@C36	100	4.3	-4.0								
G31@base···base@G32	100	4.0	-6.4	C36@base···base@C37	98	4.2	-4.2								
A34@base···base@C36	98	5.1	-3.4	C37@base···base@A38	100	4.0	-5.3								
C37@base···base@A38	100	3.9	-5.4	A38@base···base@A39	99	4.2	-5.9								
A38@base···base@A39	100	4.0	-6.1	A39@base···base@T40	98	4.2	-5.3								
A39@base···base@T40	98	4.3	-5.0												

Figure S5. D1 aptamer modelled using the APTAMD protocol. **(A)** Sequence of the random region and 2D structures predicted by mfold (*T* = 25 °C, [NaCl]=150 mM) with scoring ΔG energies in kcal/mol. **(B)** Time evolution of the RMSD computed with respect to the initial structure, and MM-PBSA energies along the cMD simulations with average and error values in kcal/mol computed for the last 1.5 µs. Evolution of the conformational entropy calculated for the last 1.5 µs of the cMD. **(C)** Superposition of the three most populated cluster representatives (ribbon model for the backbone with bases as rods) obtained by clustering analysis performed for the last 1.5 µs of the cMD simulations considering a threshold of 3 Å in the RMSD values computed for the backbone heavy atoms of nucleotides 1-40.

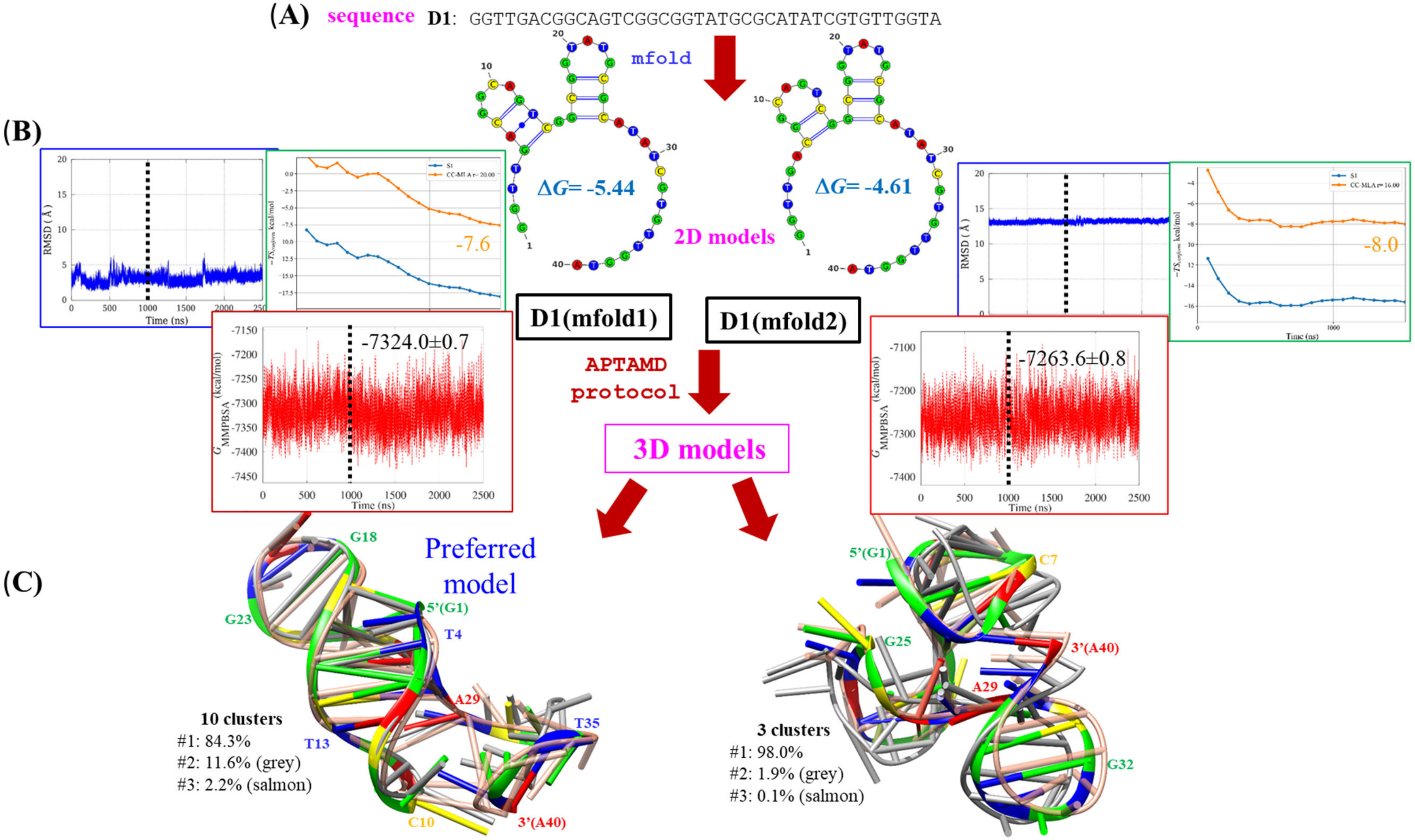

Table S5. Most abundant intramolecular H-bond contacts with percentage of occurrence (%) and average distance (d in Å) computed for the last 1.5 μs of the cMD trajectories of the collagen XI aptamers D1 and B4. The base pairing contacts in the initial 2D mfold model are in bold.

**D1(mfold1)**			**D1(mfold2)**			**B4**			**B4T$_{(11-40)}$**		
Contacts	%	d	Contacts	%	d	Contacts	%	d	Contacts	%	d
G1@N2···N3@A27	98	3.0	G2@N1···O6@G15	94	3.0	G1@N2···N3@G8	98	3.0	G1@N2···N3@G8	--	--
G2@O6···N2@G25	98	2.9	G2@N2···N7@G15	94	3.1	G1@N3···N2@G8	66	3.3	G1@N3···N2@G8	--	--
G2@N1···O2@C17	96	3.0	T3@O2···N6@A6	96	3.0	A2@N7···N6@A7	100	3.0	A2@N7···N6@A7	--	--
G2@N2H···O2@C17	95	3.0	T4@O3'···N3@T39	99	2.9	A2@N6···N1@A7	98	3.0	A2@N6···N1@A7	--	--
**G5@O6···N4@C14**	100	2.9	T4@OP1···N3@T28	99	2.8	C3@N4···N7@A6	99	3.0	C3@N4···N7@A6	--	--
**G5@N2···O2@C14**	100	2.9	T4@N3···N3@A40	93	3.1	C3@N3···N6@A6	90	3.1	C3@N3···N6@A6	--	--
**G5@N1···N3@C14**	100	3.0	**C7@N3···N1@G15**	**100**	**3.0**	T12@N3···O6@G16	--	--	T12@N3···O6@G16	79	2.9
**A6@N1···N3@T13**	100	3.0	**C7@N4···O6@G15**	**100**	**2.9**	**C14@N3···N1@G24**	77	3.0	**C14@N3···N1@G24**	100	3.0
**A6@N6···O4@T13**	100	3.0	**C7@O2···N2@G15**	**100**	**2.9**	**C14@O2···N2@G24**	77	2.9	**C14@O2···N2@G24**	99	2.9
**C7@N3···N1@G12**	100	3.0	**G8@N1···N3@C14**	**100**	**2.9**	**C14@N4···O6@G24**	76	3.0	**C14@N4···O6@G24**	98	2.9
**C7@O2···N2@G12**	100	2.9	**G8@N2···O2@C14**	**100**	**2.9**	**A15@N1···N3@T23**	100	2.9	**A15@N1···N3@T23**	100	2.9
**C7@N4···O6@G12**	100	2.9	**G8@O6···N4@C14**	**100**	**2.9**	**A15@N6···O4@T23**	97	3.0	**A15@N6···O4@T23**	97	3.0
G8@N2···N7@A11	99	3.0	G16@N3···N2@G18	100	3.0	**G16@N1···N3@C22**	100	3.0	**G16@N1···N3@C22**	100	3.0
G8@N3···N6@A11	94	3.0	**G16@N1···N3@C26**	**100**	**2.9**	**G16@N2···O2@C22**	100	2.9	**G16@N2···O2@C22**	100	2.9
C14@N4···O4@T28	97	3.0	**G16@O6···N4@C26**	**98**	**3.0**	**G16@O6···N4@C22**	100	2.9	**G16@O6···N4@C22**	100	2.9
G15@O6···N6@A27	100	2.9	**G16@N2···O2@C26**	**98**	**3.0**	**G17@N1···N3@C21**	100	3.0	**G17@N1···N3@C21**	100	3.0
G15@N1···N1@A27	100	3.0	G18@N2···OP1@T28	100	2.8	**G17@N2···O2@C21**	100	2.9	**G17@N2···O2@C21**	100	2.9

Table S5. (cont).

**D1(mfold1)**			**D1(mfold2)**			**B4**			**B4T$_{(11-40)}$**		
**G16@N2···O2@C26**	**100**	**2.9**	G18@N3···N3@T20	95	3.1	**G17@O6···N4@C21**	100	2.9	**G17@O6···N4@C21**	100	2.9
**G16@O6···N4@C26**	**99**	**2.9**	G18@N2···O3’@A27	87	3.2	A27@N6···N3@G38	--	--	A27@N6···N3@G38	100	3.0
**G16@N1···N3@C26**	**100**	**3.0**	G19@N2···O2@T20	100	2.9	A27@N7···N2@G38	--	--	A27@N7···N2@G38	99	3.0
**C17@O2···N2@G25**	**100**	**2.8**	T30@N3···O4’@T39	100	2.9	C28@OP1···N1@G37	90	2.9	**C28@N3···N1@G37**	100	2.9
**C17@N3···N1@G25**	**100**	**3.0**	T30@OP2···N1@G38	99	2.9	C28@OP1···N2@G37	89	3.0	**C28@O2···N2@G37**	100	2.9
**C17@N4···O6@G25**	**99**	**3.0**	T30@OP2···N2@G38	95	3.0	**C28@N4···O6@G37**	--	--	**C28@N4···O6@G37**	100	2.9
**G18@N1···N3@C24**	**99**	**3.0**	T30@O3’···N6@A40	89	3.0	**G29@O6···N4@C36**	99	3.0	**G29@O6···N4@C36**	100	2.9
**G18@N2···O2@C24**	**99**	**2.9**	C31@N3···N1@G37	100	3.0	**G29@N1···N3@C36**	100	2.9	**G29@N1···N3@C36**	100	3.0
**G18@O6···N4@C24**	**97**	**2.9**	C31@O2···N2@G37	100	2.9	**G29@N2···O2@C36**	100	2.8	**G29@N2···O2@C36**	100	2.9
A29@O3’···N1@G38	97	3.0	C31@N4···O6@G37	99	3.0	**A30@N6···O4@T35**	100	2.9	**A30@N6···O4@T35**	100	2.9
T30@OP1···N2@G38	97	3.0	C31@OP1···N6@A40	91	3.0	**A30@N1···N3@T35**	94	3.1	**A30@N1···N3@T35**	93	3.1
T30@OP2···N4@C31	95	2.9	G32@N2···O4@T36	91	3.0	C31@O2···N2@G34	100	2.8	C31@O2···N2@G34	100	2.8
G34@N2···O2@T35	100	2.8				C31@N3···N1@G34	100	2.9	C31@N3···N1@G34	100	2.9
T35@O4···N6@A40	98	3.0				C31@N4···O6@G34	99	2.9	C31@N4···O6@G34	100	2.9
G37@N1···OP1@A40	99	2.8				G32@N1···O4’@C36	70	3.0	G32@N1···O4’@C36	87	3.0
G37@N2··· O5’@A40	92	3.1									

Figure S6. PSAG-1 aptamer modelled using the APTAMD protocol. **(A)** Sequence for the random region of the PSAG-1 aptamer and 2D models predicted by mfold (T = 25 °C, [NaCl]=150 mM) with scoring ΔG energies in kcal/mol. **(B)** Time evolution of the MM-PBSA energies along the cMD simulations for the three models of PSAG-1 with average and error values in kcal/mol computed for the last 1.5 μs. Superposition of the three most populated cluster representatives (ribbon model for the backbone with bases as rods) obtained by clustering analysis performed for the last 1.5 μs of the cMD simulations considering a threshold of 5.5 Å in the RMSD values computed for all the backbone (i.e. sugar and phosphate) heavy atoms.

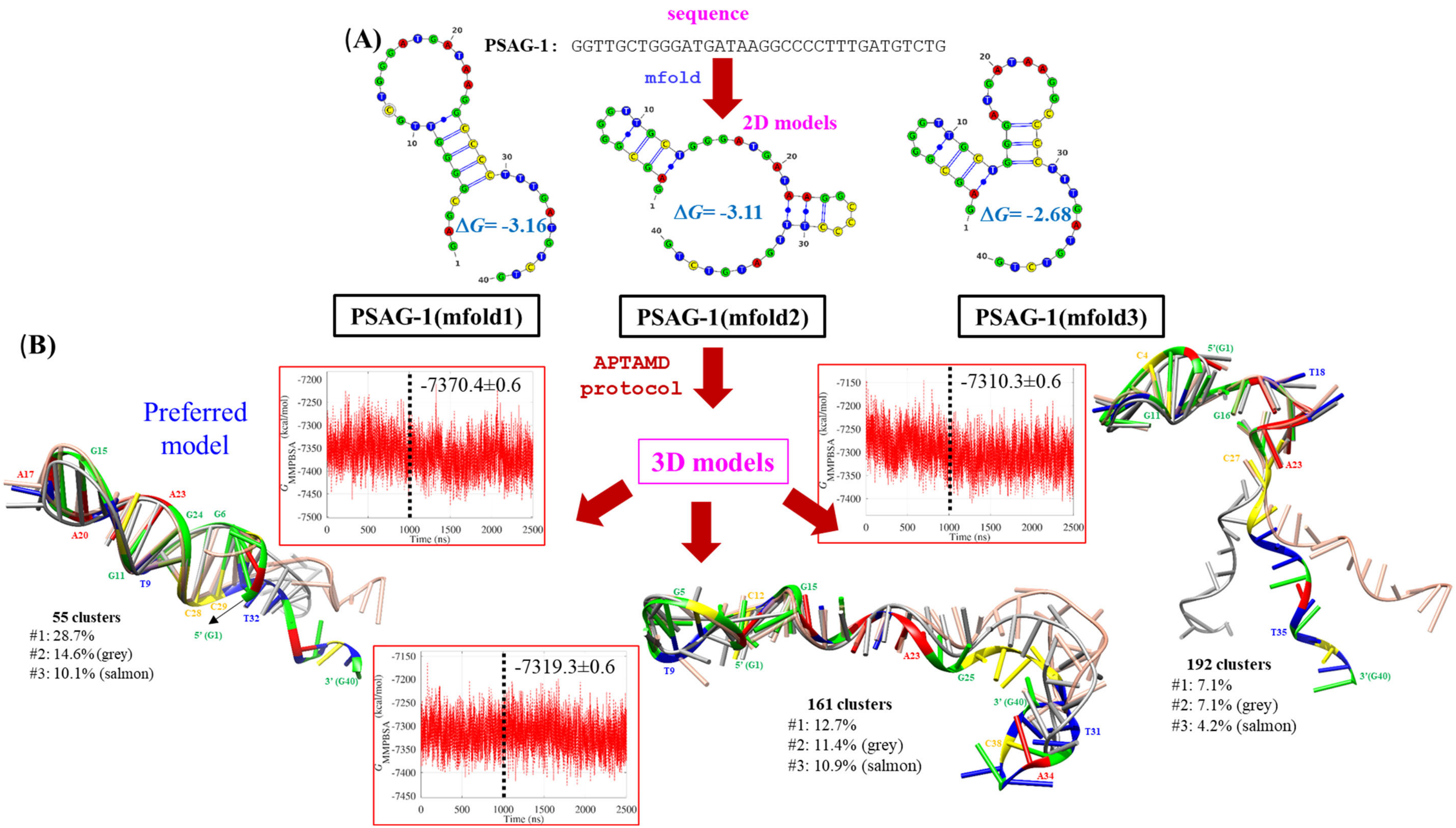

Figure S7. Sequence of the Gli1 aptamer and two versions of the truncated Gli1T(1-29) variant. 2D structures and scoring energies (kcal/mol) predicted by the mfold webserver selecting a temperature of 25 ºC and salt concentrations of 250 mM for NaCl and 5 mM for $MgCl_2$.

Gli1: C_1TAGGCGAAATATAGCTACAACTGTCTGAAGGCACCCAAT$_{40}$

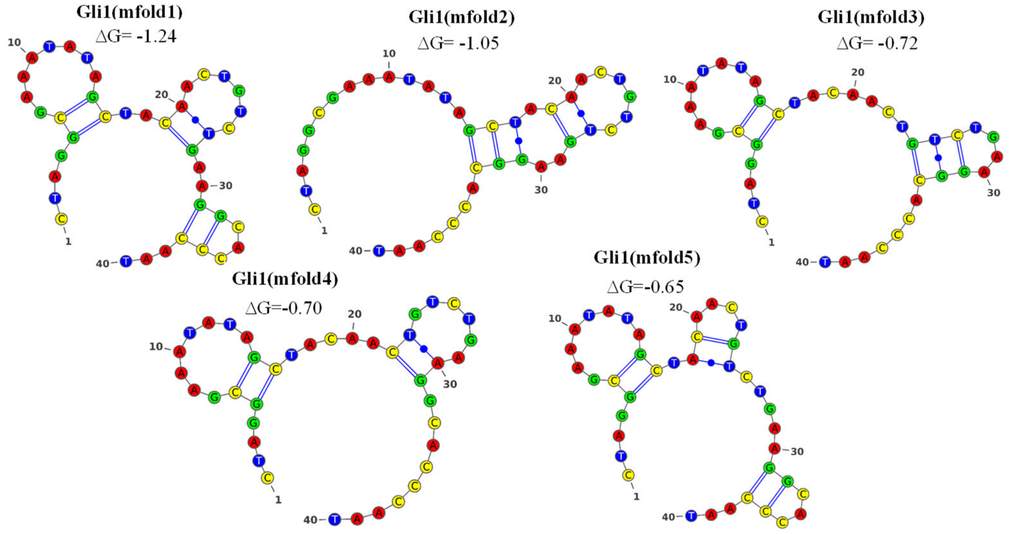

Gli1T$_{(1-29)}$: C_1TAGGCGAAATATAGCTACAACTGTCTGA$_{29}$

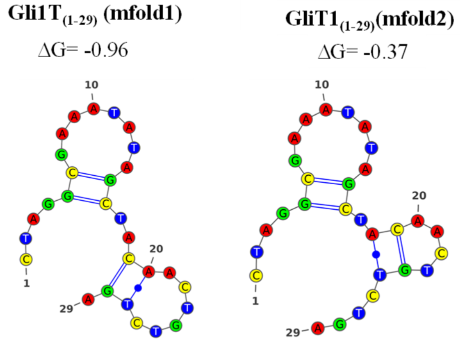

5Ts-Gli1T$_{(1-29)}$: TTTTT-C_1TAGGCGAAATATAGCTACAACTGTCTGA$_{29}$

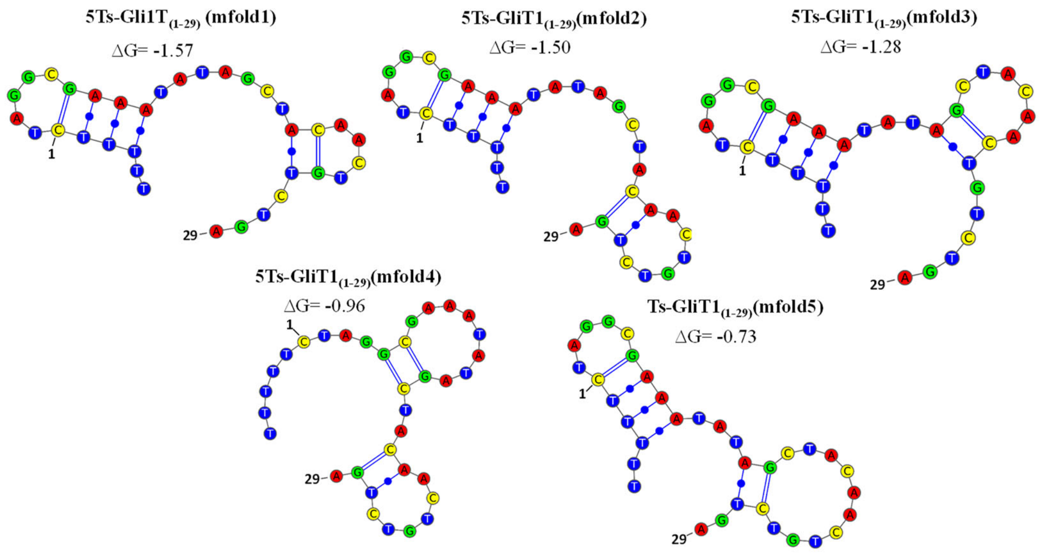

Figure S8. Sequence of the Gli4 aptamer and two versions of the truncated Gli4T$_{(22\text{-}40)}$ variant. 2D structures and scoring energies (kcal/mol) obtained with the mfold webserver selecting a temperature of 25 ºC and salt concentrations of 250 mM for NaCl and 5 mM for $MgCl_2$.

Gli4: C_1CAGTCTCCCGTTTACCGCGCCTACACATGTCTGAATGCC$_{40}$

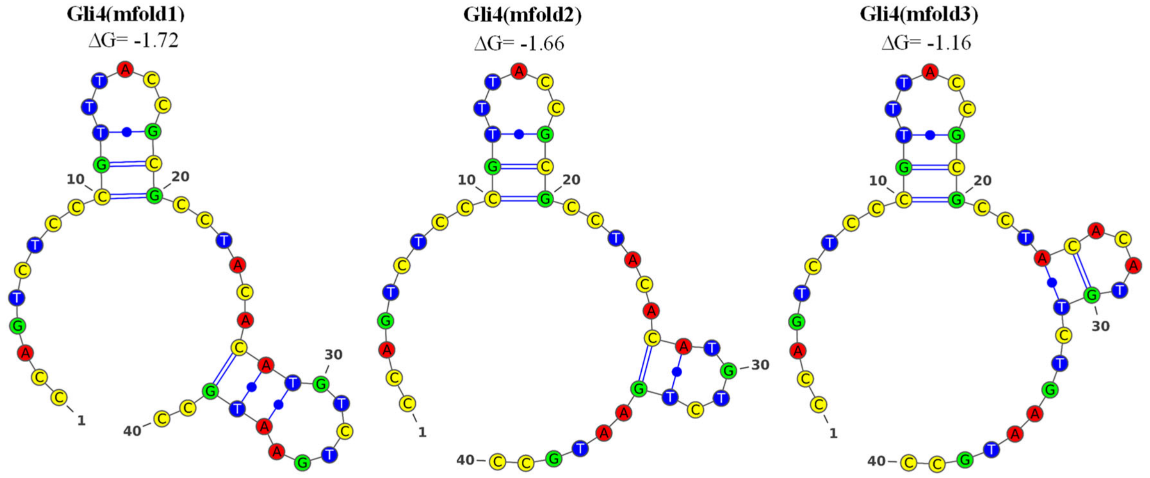

Gli4T$_{(22\text{-}40)}$: C_{22}TACACATGTCTGAATGCC$_{40}$

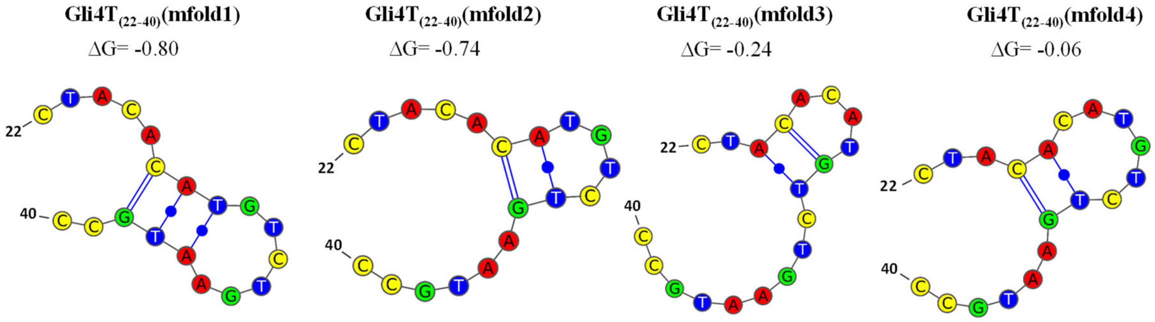

5Ts-Gli4T$_{(22\text{-}40)}$: TTTTT-C_{22}TACACATGTCTGAATGCC$_{40}$

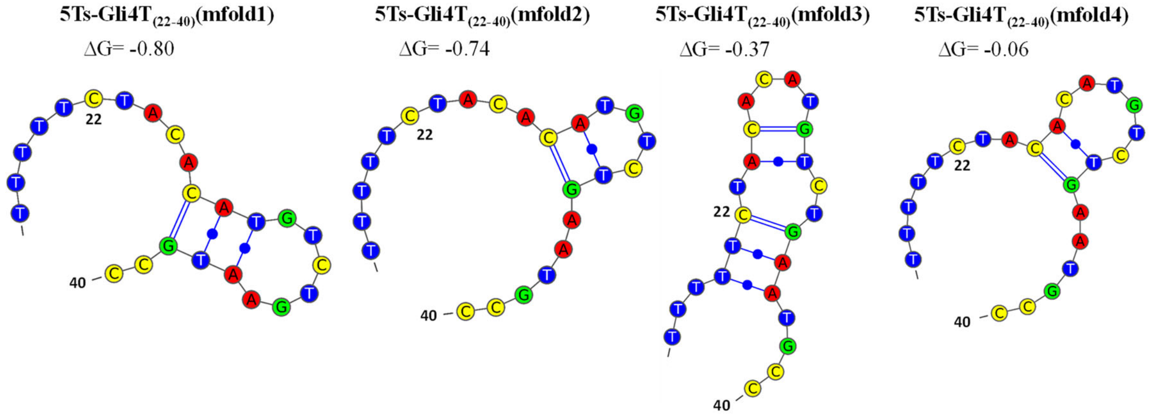

Table S6. Most abundant intramolecular H-bond contacts with percentage of occurrence (%), and average distance (d in Å) computed for the last 1.5 μs of the cMD trajectories computed for the Gli4 aptamer. The base pairing contacts in the initial 2D mfold model are in bold.

**Gli4**			**Gli4T$_{(22-40)}$**		
Polar Contacts	%	d	Polar Contacts	%	d
**C10@O2···N2@G20**	90	2.9	--	--	--
**C10@N3···N1@G20**	89	2.9	--	--	--
**C10@N4···O6@G20**	86	3.0	--	--	--
**G11@N1···N3@C19**	100	2.9	--	--	--
**G11@N2···O2@C19**	100	2.9	--	--	--
**G11@O6···N4@C19**	99	2.9	--	--	--
**T12@N3···O6@G18**	100	2.9	--	--	--
**T12@O2···N1@G18**	98	2.9	--	--	--
T13@O2···N4@C17	99	2.9	--	--	--
**C27@O2···N2@G38**	95	2.9	**C27@O2···N2@G38**	100	2.9
**C27@N3···N1@G38**	91	3.0	**C27@N3···N1@G38**	100	2.9
**C27@N4···O6@G38**	87	3.0	**C27@N4···O6@G38**	100	3.0
**A28@N1···N3@T37**	100	3.0	**A28@N1···N3@T37**	100	3.0
**A28@N6···O4@T37**	99	3.0	**A28@N6···O4@T37**	100	2.9
**T29@N3···N1@A36**	99	2.9	**T29@N3···N1@A36**	100	2.9
**T29@O4···N6@A36**	97	3.0	**T29@O4···N6@A36**	98	3.0
G30@N2···N7@A35	100	3.0	G30@N2···N7@A35	100	3.0
G30@N3···N6@A35	99	3.0	G30@N3···N6@A35	100	3.0
G30@O3'···N1@G34	97	3.1	G30@O3'···N1@G34	98	3.1
T31@OP1···N2@G34	100	2.9	T31@OP1···N2@G34	100	2.9

Table S7. Most abundant intramolecular non-polar contacts with percentage of occurrence (%), average distance (d in Å), and interaction energy (E_{int} in kcal/mol) computed for the last 1.5 μs of the cMD trajectories run for the Gli4 aptamer.

**Gli4(mfold1)**				**Gli4T$_{(22\text{-}40)}$**			
Contacts	%	d	E_{int}	Non-polar Contacts	%	d	E_{int}
C2@base···base@A3	100	4.0	-5.4	--	--	--	--
A3@base···base@G4	100	3.9	-6.6	--	--	--	--
G4@base···base@T5	100	4.2	-5.3	--	--	--	--
T5@base···base@C6	97	4.2	-4.3	--	--	--	--
C6@base···base@T7	98	4.2	-4.4	--	--	--	--
T7@base···base@C8	96	4.8	-3.5	--	--	--	--
C8@base···base@C9	93	4.4	-3.8	--	--	--	--
C9@base···base@C10	99	4.2	-4.2	--	--	--	--
C10@base···base@G11	98	4.8	-3.3	--	--	--	--
G11@base···base@T12	100	3.6	-6.5	--	--	--	--
G11@base···base@G20	100	4.9	-5.0	--	--	--	--
T12@base···base@T13	100	3.6	-5.4	--	--	--	--
T13@base···base@T14	100	3.8	-5.1	--	--	--	--
T14@base···base@A15	99	5.3	-3.3	--	--	--	--
C17@base···base@G18	100	4.0	-5.7	--	--	--	--
G18@base···base@C19	100	3.6	-6.3	--	--	--	--
C19@base···base@G20	100	4.3	-4.3	--	--	--	--
G20@base···base@C21	93	5.0	-4.3	--	--	--	--
C21@base···base@G22	99	4.4	-3.9	--	--	--	--
C22@base···base@T23	99	4.3	-4.3	C22@base···base@T23	100	4.0	-3.5
T23@base···base@A24	90	4.3	-5.0	T23@base···base@A24	99	4.0	-5.5
A24@base···base@C25	91	4.4	-4.6	A24@base···base@C25	100	4.1	-4.8
C25@base···base@A26	100	4.0	-5.3	C25@base···base@A26	100	3.9	-5.6
A26@base···base@C27	91	4.6	-4.4	A26@base···base@C27	100	3.6	-5.9
C27@base···base@A28	100	4.6	-3.5	C27@base···base@A28	100	4.5	-3.5
A28@base···base@T29	100	3.7	-6.3	A28@base···base@T29	100	3.6	-6.3
A28@base···base@G38	100	4.8	-4.9	A28@base···base@G38	100	5.2	-4.3
T29@base···base@G30	100	3.8	-6.1	T29@base···base@G30	100	3.8	-6.2
G30@base···base@G34	100	4.4	-5.4	G30@base···base@G34	100	4.4	-5.3
C32@base···base@T33	100	4.4	-3.8	C32@base···base@T33	100	4.0	-4.0
T33@base···base@G34	100	4.2	-5.4	T33@base···base@G34	100	4.2	-5.2
G34@base···base@A35	100	5.0	-4.2	G34@base···base@A35	100	4.9	-4.2
A35@base···base@A36	100	3.8	-6.5	A35@base···base@A36	100	3.8	-6.5
A36@base···base@T37	100	3.9	-5.4	A36@base···base@T37	100	4.1	-4.9
T37@base···base@G38	100	4.6	-4.0	T37@base···base@G38	100	3.9	-5.6
G38@base···base@C39	99	4.7	-4.1	G38@base···base@C39	100	5.5	-2.5

Figure S9. Time evolution of the root mean squared deviation (RMSD in Å) for all the heavy atoms in selected residues with respect to the initial structure and MM-PBSA energy values (kcal/mol) computed along the cMD simulations of the PSA-1 aptamer.

a) **PSA-1**

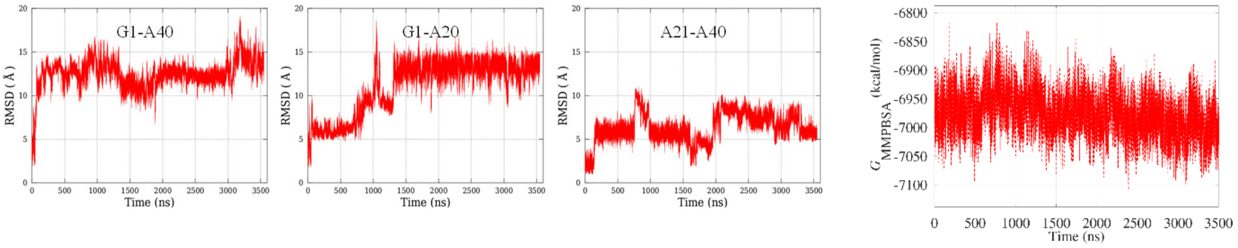

b) **PSA-1T$_{(15\text{-}40)}$**

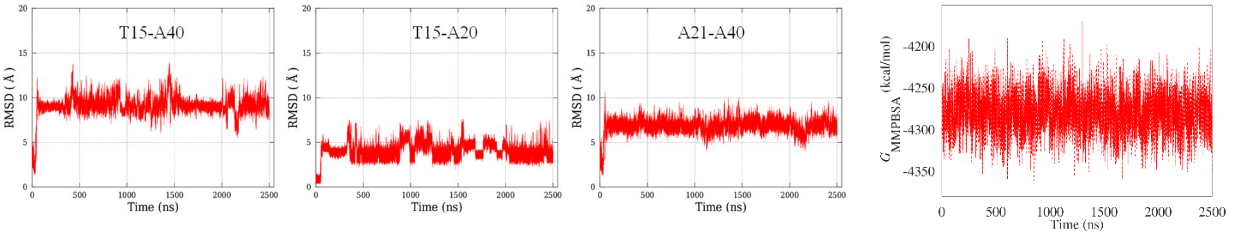

Figure S10. Sequence of the PSA-1 and PSA-1(15-40) aptamers. 2D structures and scoring energies obtained with the mfold webserver selecting a temperature of 25 ºC and salt concentrations of 300 mM for NaCl.

PSA-1: G_1GACGGTTGCGCTATATTTAACCAAAAGTCTGGATTAACA$_{40}$

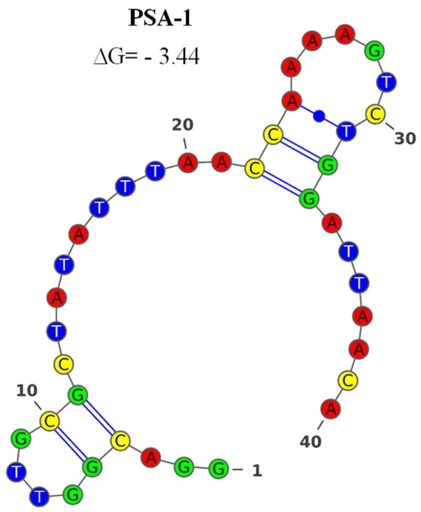

PSA-1T(15-40): T_{15}ATTTAACCAAAAGTCTGGATTAACA$_{40}$

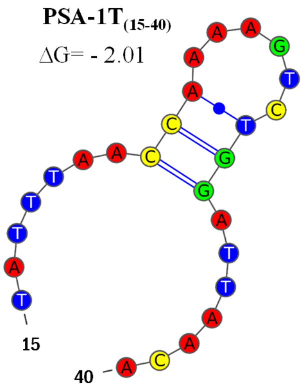

Table S8. Most abundant intramolecular H-bond contacts with percentage of occurrence (%) and average distance (d in Å) computed for the last 1.5 μs of the cMD trajectories run for the PSA-1 aptamer. The base pairing contacts in the initial 2D mfold model are in bold.

**PSA-1**			**PSA-1T$_{(15-40)}$**		
Contact	%	d	Contact	%	d
G6@N2···O2@T7	90	3.0	A21OP1···N4@C22	76	2.9
T17@OP2···N3@T19	74	2.9	A21N7···N6@A37	52	3.2
**C22@O2···N2@G33**	99	2.9	C22@O4'···N3@T36	85	2.9
**C22@N3···N1@G33**	99	3.0	**C22@O2···N2@G33**	100	2.9
**C22@N4···O6@G33**	97	3.0	**C22@N3···N1@G33**	100	3.0
**C23@O2···N2@G32**	100	2.8	**C22@N4···O6@G33**	97	2.9
**C23@N3···N1@G32**	100	3.0	**C23@O2···N2@G32**	100	2.9
**C23@N4···O6@G32**	99	2.9	**C23@N3···N1@G32**	100	3.0
**A24@N6···O4@T31**	98	2.9	**C23@N4···O6@G32**	99	2.9
**A24@N1···N3@T31**	98	3.0	**A24@N6···O4@T31**	97	2.9
G28@N2···O2@T29	95	2.9	**A24@N1···N3@T31**	97	3.0
			G28@N2···O2@T29	95	2.9

Table S9. Most abundant non-polar intramolecular contacts with percentage of occurrence (%), average distance (d in Å) and average interaction energy (E_{int}) computed for the last 1.5 μs of the cMD trajectories of the PSA-1 aptamer.

**PSA-1**				**PSA-1T$_{(15-40)}$**			
Contact	%	d	E_{int}	Contact	%	d	E_{int}
G1@base···base@G2	100	4.1	-5.0	--	--	--	--
G2@base···base@A3	100	4.1	-6.2	--	--	--	--
A3@base···base@C4	100	4.1	-5.0	--	--	--	--
C4@base···base@G5	100	3.9	-5.7	--	--	--	--
G5@base···base@G6	100	4.1	-6.2	--	--	--	--
G6@base···base@T8	100	4.6	-4.2	--	--	--	--
T8@base···base@G9	98	4.2	-4.9	--	--	--	--
G9@base···base@C10	100	4.0	-5.4	--	--	--	--
G10@base···base@G11	100	4.0	-5.6	--	--	--	--
G11@base···base@C12	100	4.2	-5.0	--	--	--	--
C12@base···base@T13	100	3.9	-4.8	--	--	--	--
T13@base···base@A14	100	4.0	-5.5	--	--	--	--
A14@base···base@T15	99	4.0	-5.4	--	--	--	--
T15@base···base@A16	100	4.0	-5.5	T15@base···base@A16	100	3.9	-4.4
A16@base···base@T17	100	4.1	-5.4	A16@base···base@T17	99	4.1	-5.3
T17@base···base@T19	100	5.8	-1.7	T17@base···base@T18	72	4.3	-4.6
T18@base···base@A38	57	4.7	-4.3	T18@base···base@T19	97	4.3	-4.4
T19@base···base@A20	100	4.0	-5.4	T19@base···base@A20	98	4.0	-5.3
A20@base···base@A21	100	4.1	-5.9	A20@base···base@A21	100	4.2	-5.6
A21@base···base@C22	99	5.1	-4.0	A21@base···base@A37	100	5.0	-4.1
C22@base···base@C23	100	4.0	-4.4	C22@base···base@C23	100	4.1	-4.2
C23@base···base@A24	100	4.4	-3.8	C23@base···base@A24	100	4.4	-3.7
A24@base···base@A25	100	4.0	-6.4	A24@base···base@A25	100	3.9	-6.4
A24@base···base@G32	100	5.3	-4.1	A24@base···base@G32	100	5.3	-4.2
A25@base···base@A26	100	3.8	-6.6	A25@base···base@A26	100	3.8	-6.6
A26@base···base@A27	100	3.9	-6.4	A26@base···base@A27	99	4.0	-6.3
G28@base···base@C30	100	4.4	-4.1	G28@base···base@C30	100	4.4	-4.1
T31@base···base@G32	100	4.3	-4.6	T31@base···base@G32	100	4.3	-4.6
G32@base···base@G33	100	4.1	-6.4	G32@base···base@G33	100	4.1	-6.5
G33@base···base@A34	95	4.4	-5.8	G33@base···base@A34	100	3.7	-7.0
A34@base···base@T35	100	4.0	-5.5	A34@base···base@T35	100	3.8	-5.7
T35@base···base@T36	57	4.6	-4.0	T36@base···base@A37	100	4.0	-5.1
T36@base···base@A37	90	4.6	-4.6	A37@base···base@A38	95	4.9	-4.6
A37@base···base@A38	99	4.3	-5.6	A38@base···base@C39	97	4.9	-4.0
C39@base···base@A40	93	4.9	-4.2	C39@base···base@A40	87	4.2	-4.9

Figure S11. Superposition of the three most populated cluster representatives obtained from cluster calculations performed for the last 1.5 μs of the simulations and considering a threshold of 5.5 Å in the RMSD values computed for the backbone (i.e. sugar and phosphate) heavy atoms of all nucleotides.

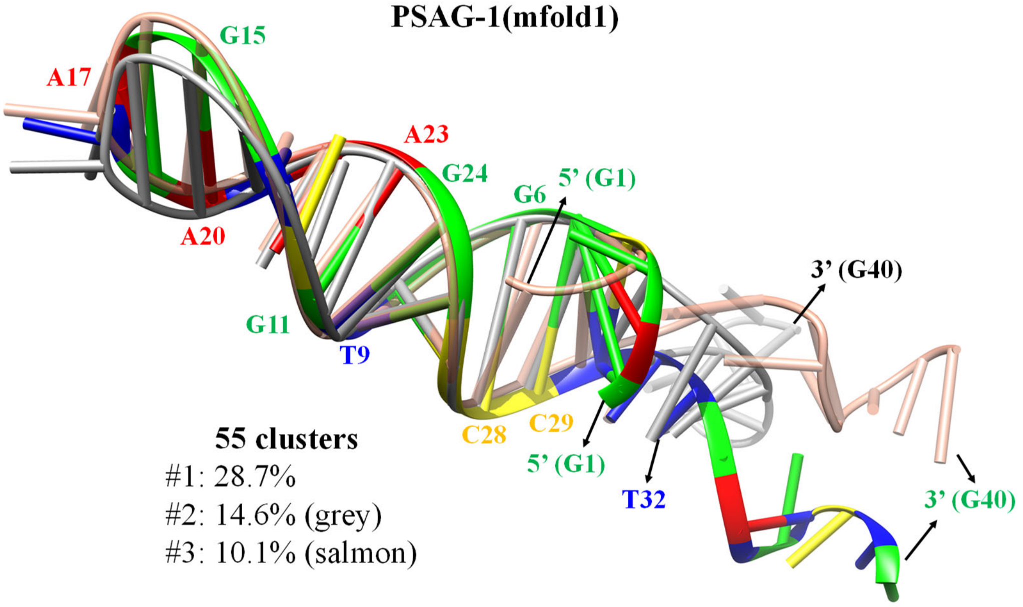

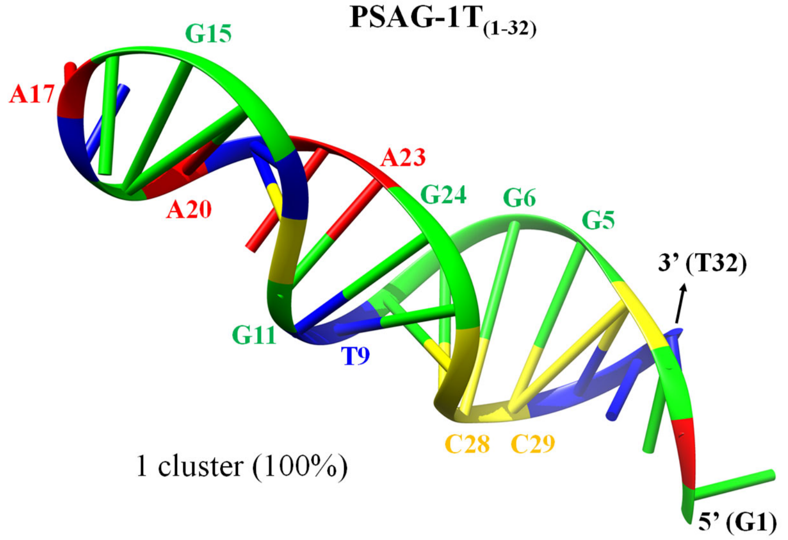

Figure S12. Sequence of **D1** and **B4** and **B4T$_{(11\text{-}40)}$** aptamers selected against a collagen XI peptide with conserved motifs highlighted in blue and grey. 2D structures and scoring energies obtained with the mfold webserver selecting a temperature of 25 ºC and a NaCl salt concentration of 150 mM.

D1: G$_{1}$GTTGACGGCAGTCGGCGGTATGCGCATATCGTATTGGTA$_{40}$

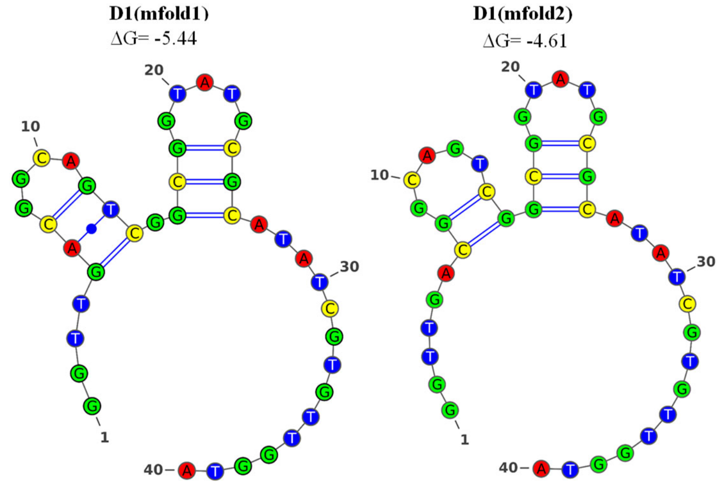

B4: G$_{1}$ACGCAAGAAATTCAGGGCACCTGGAACGACGCGTCGGCT$_{40}$

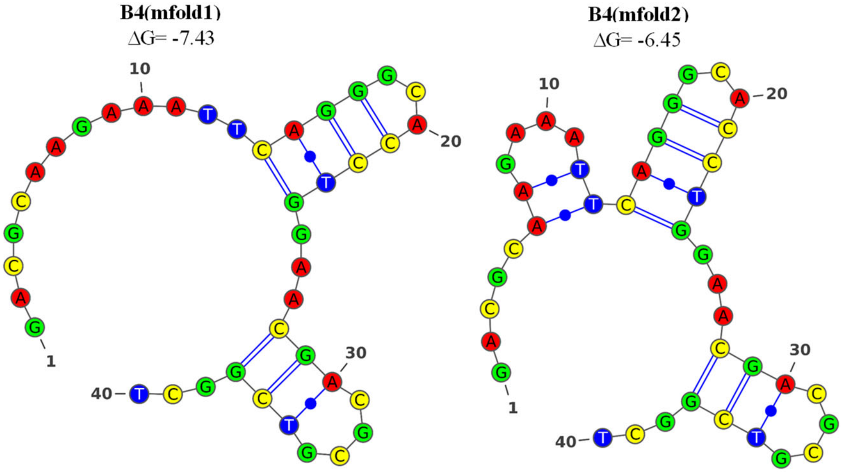

B4T$_{(11\text{-}40)}$: A$_{11}$TTCAGGGCACCTGGAACGACGCGTCGGCT$_{40}$

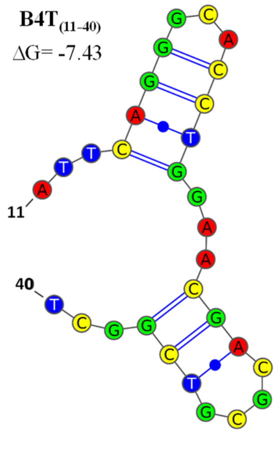

Figure S13. Time evolution of the root mean squared deviation (RMSD in Å) for all the heavy atoms in selected residues with respect to the initial structure and MM-PBSA energy values (kcal/mol) computed along the cMD simulations run for the collagen XI aptamers **D1** and **B4**.

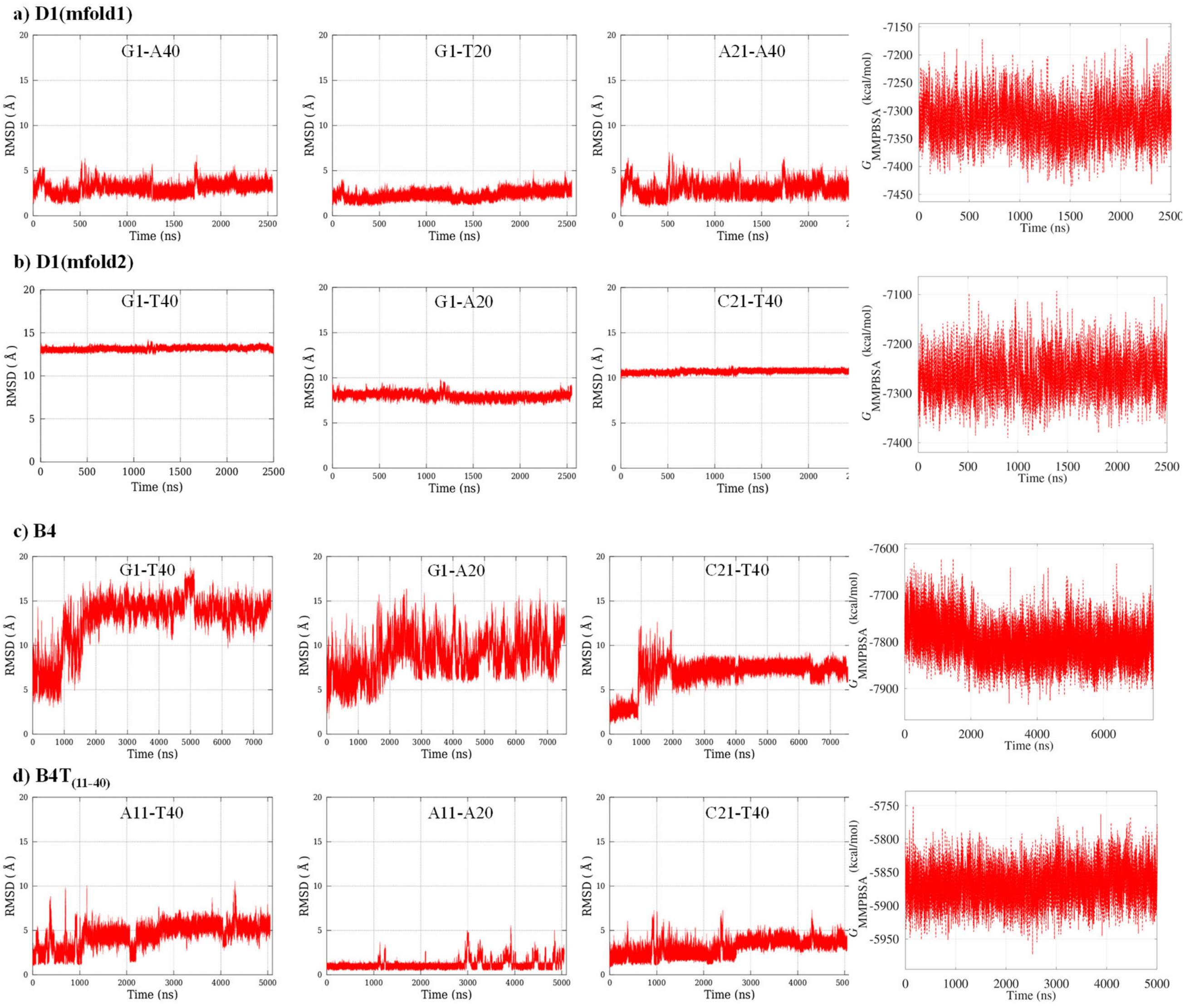

Table S10. Most abundant (percentage of occurrence > 90%) non-polar intramolecular contacts with average distance (d in Å) and average interaction energy (E_{int}) computed for the last 1.5 μs of the cMD trajectories of the collagen XI aptamer D1 and B4.

**D1(mfold1)**			**D1(mfold2)**			**B4**			**B4T(11-40)**		
Contact	d	E_{int}	Contact	d	E_{int}	Contact	d	E_{int}	Contact	d	E_{int}
T3@base···base@T4	3.8	-5.3	G1@base···base@G2	4.4	-4.5	G1@base···base@A2	4.0	-5.3	--	--	--
T4@base···base@G5	3.9	-5.8	G2@base···base@T3	3.7	-6.4	A2@base···base@C3	4.1	-4.7	--	--	--
G5@base···base@A6	4.1	-6.2	T4@base···base@G5	3.9	-5.2	G4@base···base@C5	4.0	-5.8	--	--	--
A6@base···base@C7	3.9	-5.6	T4@base···base@T30	4.4	-4.0	C5@base···base@A6	3.8	-5.5	--	--	--
C7@base···base@G8	3.8	-5.9	G5@base···base@A6	3.5	-7.8	A6@base···base@A7	3.7	-7.0	--	--	--
G8@base···base@G9	4.2	-6.2	A6@base···base@C7	3.6	-5.8	A7@base···base@G8	4.1	-6.1	--	--	--
A11@base···base@G12	3.8	-7.0	A6@base···base@G15	5.8	-3.1	G8@base···base@A9	3.8	-6.8	--	--	--
G12@base···base@T13	3.8	-6.2	C7@base···base@G8	4.5	-3.2	A9@base···base@A10	4.0	-6.0	--	--	--
T13@base···base@C14	4.4	-3.9	G8@base···base@G9	3.8	-7.1	A10@base···base@A11	4.0	-6.1	--	--	--
C14@base···base@G15	3.6	-6.0	G8@base···base@G15	5.9	-3.3	A11@base···base@A20	--	--	A11@base···base@A20	4.2	-5.2
G15@base···base@G16	3.9	-6.7	C10@base···base@A11	4.1	-5.0	A11@base···base@T12	4.0	-5.5	A11@base···base@T12	4.1	-4.4
G16@base···base@C17	3.6	-6.0	A11@base···base@G12	3.9	-6.6	T12@base···base@T13	4.7	-4.1	T12@base···base@A15	5.2	-3.3
C17@base···base@G18	4.8	-3.0	C14@base···base@G15	3.8	-5.2	C14@base···base@T40	5.3	-3.5	T13@base···base@T40	5.0	-3.4
G18@base···base@G19	4.0	-6.6	G16@base···base@A27	5.0	-3.6	C14@base···base@A15	4.8	-3.1	C14@base···base@A15	4.0	-4.8
G18@base···base@G25	5.0	-4.8	G18@base···base@G19	3.9	-7.0	A15@base···base@G16	3.8	-6.8	A15@base···base@G16	4.3	-5.5
G19@base···base@T20	4.1	-5.4	G19@base···base@A21	4.2	-5.9	A15@base···base@G24	5.3	-4.1	A15@base···base@G24	5.3	-4.0
T20@base···base@A21	4.1	-5.0	T22@base···base@G23	4.6	-4.5	C16@base···base@G17	4.1	-6.2	G16@base···base@G17	3.8	-6.8

Table S10. (cont).

D1(mfold1)			D1(mfold2)			B4			B4T(11-40)		
T22@base···base@G23	3.8	-5.8	G25@base···base@C26	3.7	-6.0	G17@base···base@C19	4.0	-5.0	G17@base···base@C19	4.0	-5.3
G23@base···base@C24	5.2	-3.4	C26@base···base@A27	4.1	-5.3	G17@base···base@G18	5.2	-3.4	G17@base···base@G18	5.5	-3.0
C24@base···base@G25	4.2	-4.3	A27@base···base@T28	4.0	-5.5	C21@base···base@C22	3.8	-4.8	C21@base···base@C22	3.8	-4.8
G25@base···base@C26	4.0	-5.8	T28@base···base@A29	3.7	-6.2	C22@base···base@T23	4.0	-4.1	C22@base···base@T23	3.7	-5.0
C26@base···base@A27	4.6	-3.3	T30@base···base@G38	4.9	-4.3	T23@base···base@G24	4.3	-4.6	T23@base···base@G24	4.4	-4.2
A27@base···base@T28	4.4	-4.4	C31@base···base@G32	3.6	-6.2	G24@base···base@G25	4.1	-6.1	G24@base···base@G25	3.8	-7.0
A29@base···base@T30	4.0	-5.4	G32@base···base@T33	4.2	-5.1	G25@base···base@A26	4.3	-5.6	G25@base···base@A26	4.1	-5.9
C31@base···base@G32	3.7	-5.9	G34@base···base@T35	3.7	-6.1	A26@base···base@A27	4.0	-6.0	A26@base···base@G38	4.2	-6.4
G32@base···base@T35	3.9	-6.0	T35@base···base@T36	3.8	-5.3	A27@base···base@C28	--	--	A27@base···base@C28	3.9	-4.9
G34@base···base@A40	3.8	-7.1	T36@base···base@G37	3.8	-5.9	A27@base···base@G37	4.1	-5.8	A27@base···base@G37	5.6	-3.4
G34@base···base@T36	4.7	-3.6	G37@base···base@G38	3.8	-6.8	C28@base···base@G37	5.7	-2.3	C28@base···base@G29	4.7	-3.0
G37@base···base@G38	3.7	-7.0				G29@base···base@A30	3.8	-6.8	G29@base···base@A30	3.8	-7.0
G38@base···base@T39	5.0	-3.0				G29@base···base@G37	5.2	-4.1	G29@base···base@G37	5.8	-3.5
						A30@base···base@C31	3.7	-5.8	A30@base···base@C31	3.6	-6.0
						G31@base···base@C33	4.2	-4.2	C31@base···base@C33	4.1	-4.2
						G34@base···base@T35	3.6	-6.4	G34@base···base@T35	3.6	-6.5
						T35@base···base@C36	4.0	-4.6	T35@base···base@C36	4.4	-4.0
						C36@base···base@G37	4.0	-5.3	C36@base···base@G37	3.8	-5.2
						G37@base···base@G38	--	--	G37@base···base@G38	4.3	-5.7
						C39@base···base@T40	4.4	-4.0	C39@base···base@T40	--	--

Figure S14. Superposition of the three most populated cluster representatives obtained from cluster calculations performed for the last 2.5 μs of the B4 and B4T simulations considering a threshold of 3.0 Å (5.5 for B4) in the RMSD values computed for the backbone (i.e. sugar and phosphate) heavy atoms of all nucleotides.

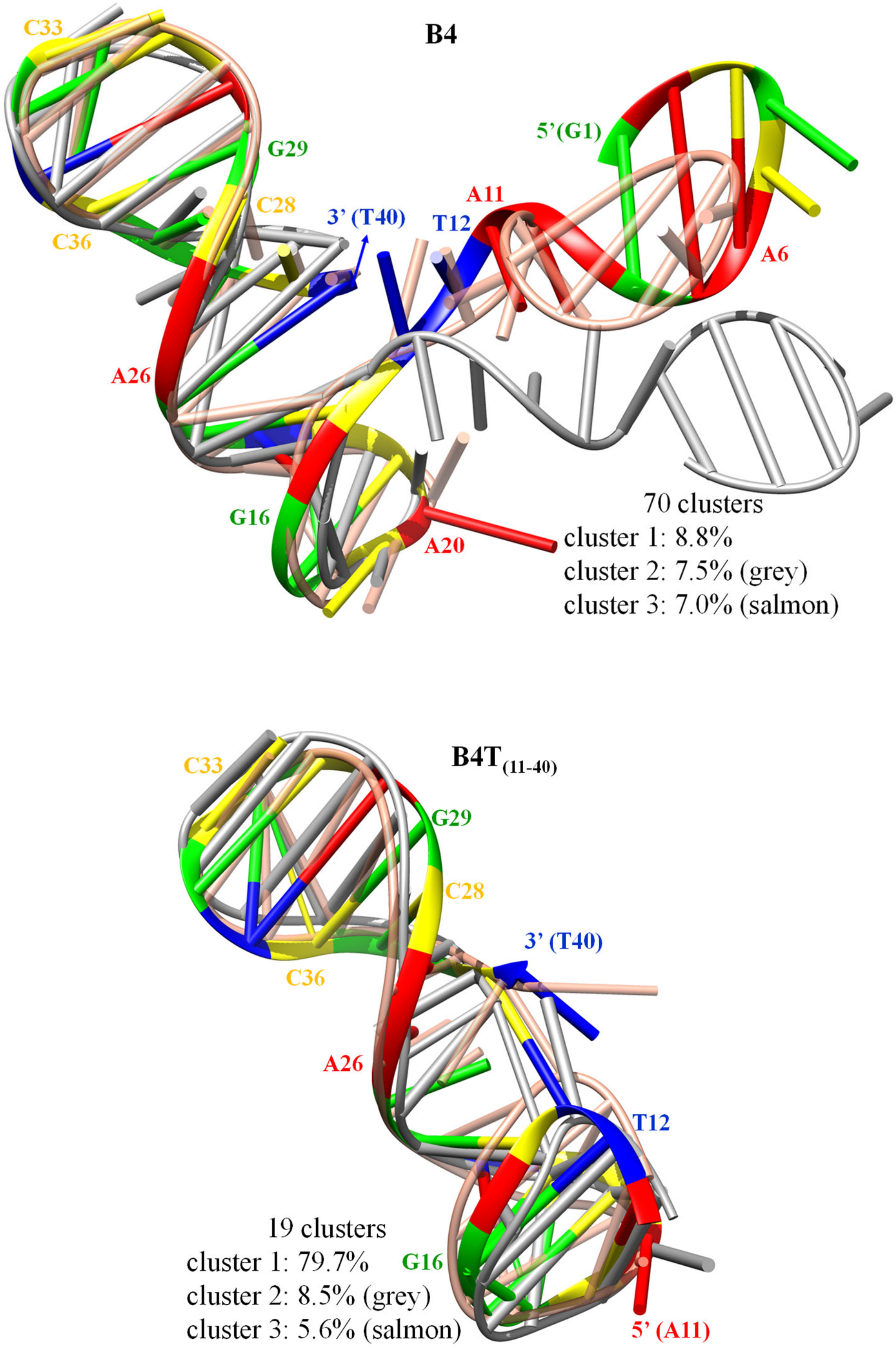

Figure S15. Time evolution of the root mean squared deviation (RMSD in Å) for all the heavy atoms in selected residues with respect to the initial structure and MM-PBSA energy values (kcal/mol) computed along the cMD simulations of the anti-PSA 24nt aptamers.

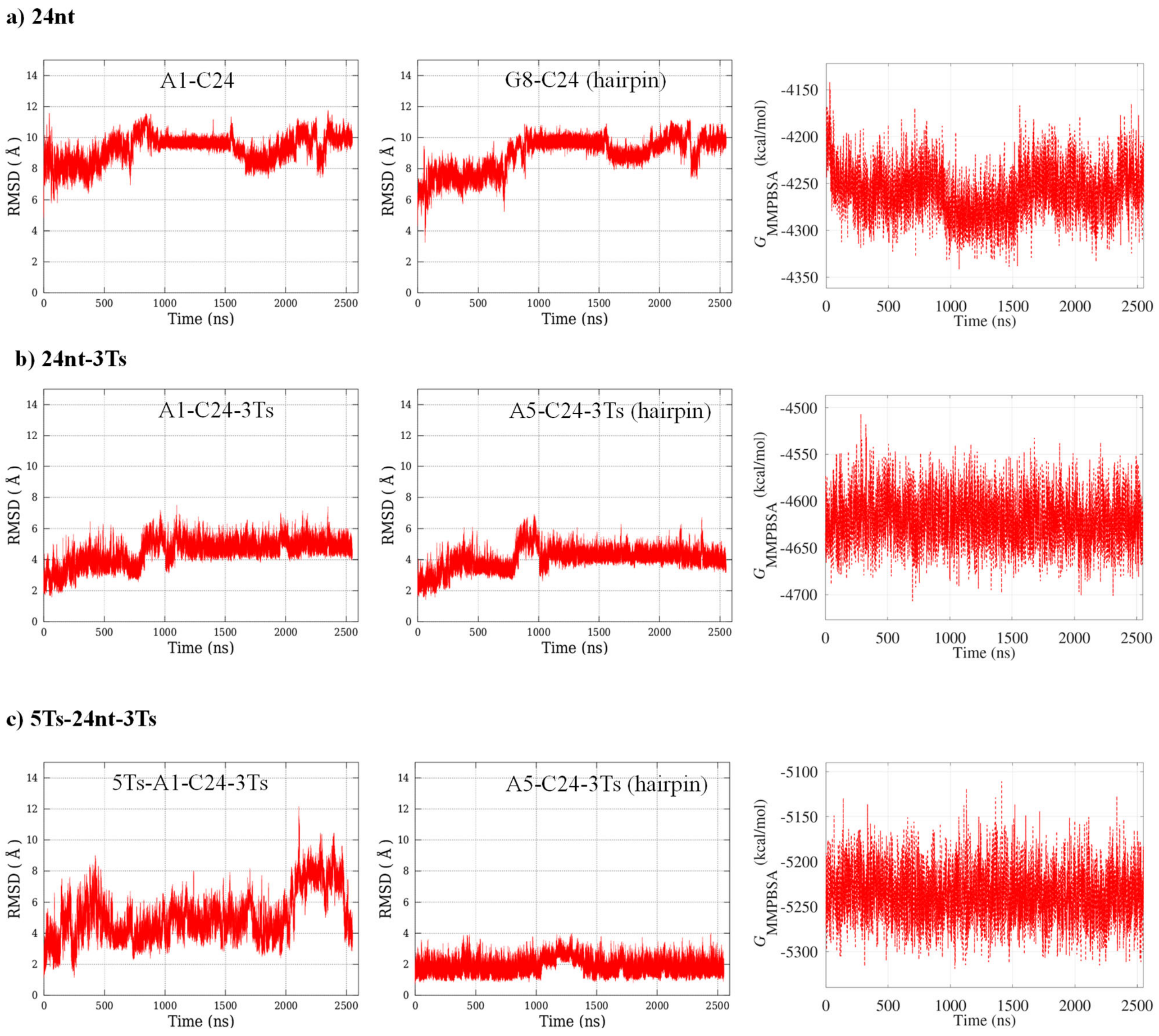

Table S11. Most abundant intramolecular H-bond contacts with percentage of occurrence (%) and average distance (d in Å) computed for the last 1.5 μs of the cMD trajectories run for different variants of the anti-PSA 24nt aptamer. The base pairing contacts in the initial 2D mfold model are in bold.

	**24nt**		**24nt-3Ts**		**5Ts-24nt-3Ts**	
Contact	%	d	%	d	%	d
**A5@N1···N3@T27**	--	--	80	3.0	31	3.0
**A5@N6···O4@T27**	--	--	78	3.0	30	3.0
**A6@N1···N3@T26**	--	--	98	3.0	94	3.0
**A6@N6···O4@T26**	--	--	97	3.0	92	3.0
**A7@N1···N3@T25**	--	--	100	2.9	100	2.9
**A7@N6···O4@T25**	--	--	98	3.0	97	3.0
**G8@N2···O2@C24**	100	2.9	100	2.9	100	2.9
**G8@N1···N3@C24**	100	3.0	100	2.9	100	2.9
**G8@O6···N4@C24**	98	3.0	100	3.0	100	2.9
**C9@N3···N1@G23**	100	3.0	100	3.0	100	3.0
**C9@O2···N2@G23**	100	2.9	100	2.8	100	2.9
**C9@N4···O6@G23**	99	3.0	99	3.0	100	2.9
**T10@N3···N1@A22**	63	2.9	100	2.9	99	2.9
**T10@O4···N6@A22**	59	3.0	84	3.1	97	3.0
C11@N4···O4@T21	22	3.0	100	2.9	100	2.9
C11@N3···N3@T21	18	3.0	97	3.0	100	3.0

Table S12. Most abundant non-polar intramolecular contacts with percentage of occurrence (%), average distance (d in Å) and average interaction energy (E_{int}) computed for the last 1.5 μs of the cMD trajectories run for different variants of the anti-PSA 24nt aptamer.

	**24nt**			**24nt-3Ts**			**5Ts-24nt-3Ts**		
Contact	%	d	E_{int}	%	d	E_{int}	%	d	E_{int}
T-5@base···base@T-4	--	--	--	--	--	--	100	4.0	-3.6
T-4@base···base@T-3	--	--	--	--	--	--	99	4.0	-4.9
T-3@base···base@T-2	--	--	--	--	--	--	26	5.2	-3.4
T-2@base···base@T-1	--	--	--	--	--	--	100	4.0	-4.7
T-1@base···base@A1	--	--	--	--	--	--	100	4.1	-5.4
A1@base···base@A2	99	4.1	-5.0	99	4.2	-4.9	100	3.9	-6.4
A2@base···base@T3	100	3.7	-6.1	98	5.0	-3.4	98	4.0	-5.5
T3@base···base@T4	50	4.6	-4.2	93	6.7	-1.8	95	4.4	-4.4
T4@base···base@A5	100	3.9	-5.6	98	4.1	-5.0	99	4.0	-5.3
A5@base···base@A6	100	4.0	-6.0	100	4.0	-6.2	100	4.0	-6.1
A6@base···base@A7	100	4.0	-6.1	100	3.8	-6.7	100	3.9	-6.5
A7@base···base@G8	100	3.8	-7.0	100	4.1	-6.0	100	4.2	-5.8
G8@base···base@C9	100	3.9	-5.8	100	3.6	-6.4	100	3.6	-6.4
C9@base···base@T10	100	3.9	-4.8	100	3.7	-5.0	100	3.9	-4.5
T10@base···base@C11	88	5.0	-3.2	100	4.3	-4.1	100	3.7	-5.2
C11@base···base@G12	52	7.3	-1.0	100	3.9	-5.1	100	5.2	-3.2
G12@base···base@C13	100	3.9	-5.7	97	6.3	-1.8	100	6.6	-1.8
G12@base···base@A19	72	6.8	-1.6	97	4.8	-5.0	100	7.5	-0.3
G12@base···base@A20	76	6.2	-2.7	88	7.5	-1.3	100	4.3	-5.3
G12@base···base@T21	74	7.2	-0.8	100	5.3	-3.1	100	4.2	-5.0
C13@base···base@C14	100	4.2	-4.1	55	6.6	-1.6	100	3.9	-4.4
C14@base···base@A15	100	4.1	-5.2	75	6.8	-0.7	100	3.8	-5.8
A15@base···base@T16	31	4.3	-5.0	100	3.8	-5.9	6	9.4	-0.1
T16@base···base@C17	95	4.2	-4.3	100	4.1	-4.4	100	3.8	-4.8
C17@base···base@A18	87	4.6	-4.6	100	4.0	-5.4	100	4.1	-5.3
A18@base···base@A19	99	4.0	-6.4	100	3.9	-6.5	100	4.0	-6.2
A19@base···base@A20	100	4.8	-4.7	88	6.2	-3.0	100	3.7	-6.9
A20@base···base@T21	100	3.9	-5.7	98	7.2	-1.0	97	7.9	-0.2
T21@base···base@A22	87	5.1	-4.0	100	3.8	-5.5	100	4.1	-4.7
A22@base···base@G23	100	4.0	-6.3	100	4.3	-5.2	100	4.0	-6.2
G23@base···base@C24	100	3.8	-6.2	100	3.7	-6.3	100	3.7	-6.2
C24@base···base@T25	--	--	--	100	3.9	-4.6	100	3.9	-4.6
T25@base···base@T26	--	--	--	100	4.0	-4.8	100	3.9	-5.1
T26@base···base@T27	--	--	--	100	4.0	-5.1	81	4.2	-4.9
